# Thinking-aloud methodology applied to health questionnaire development and validation: a scoping review

**DOI:** 10.1186/s12874-026-02927-9

**Published:** 2026-06-20

**Authors:** Laura Tasson, Marta Bonato, Farah D’Odorico, Matteo Danielis, Matteo Martinato, Cristina Canova

**Affiliations:** 1 Program in Translational Specialistic Medicine “G.B. Morgagni”, Curriculum “Nursing and Health Sciences, University of Padua, Padua, Italy; 2 Chief Nurse Office, Azienda Unita Locale Socio Sanitaria (AULSS) 6 Euganea, Padua, Italy; 3 Unit of Biostatistics, Epidemiology and Public Health, Department of Cardio-Thoraco-Vascular Sciences and Public Health, University of Padua, Padua, Italy; 4 Laboratory of Studies and Evidence Based Nursing, Department of Cardiac, Thoracic, Vascular Sciences and Public Health, University of Padua, Via Loredan 18, Padua, 35131 Italy

**Keywords:** Thinking-aloud, Cognitive interviewing, Questionnaire validation, Scoping review

## Abstract

**Background:**

The development of valid and reliable health questionnaires relies on respondents’ accurate comprehension of items. The thinking-aloud (TA) technique, where participants verbalize their thought processes while answering survey questions, offers a powerful means to explore cognitive mechanisms underlying response behaviour. Despite its wide use, there is no consensus on how TA protocols should be designed, conducted, and analysed.

**Objective:**

This scoping review aimed to map methodological approaches applied to TA in health-related questionnaire development and validation, describe their variability, and critically examine current methodological practices to inform future research.

**Design:**

PubMed, Scopus, and Embase were searched on July 17, 2025, without time limits. Eligible studies were original articles in English applying TA for questionnaire validation, including assessment of content, face, construct validity, or comprehensibility. Two reviewers independently screened records and extracted data using a standardized charting form. An operational criterion was used to judge the completeness of methodological reporting. Data were synthesized narratively and tabulated to describe study purposes, TA typologies, analytical strategies, and theoretical frameworks.

**Results:**

From 1,678 retrieved records, 210 studies met inclusion criteria, of which 84 provided complete methodological descriptions. Most of the 84 studies involved patients (48%) and used concurrent TA (48%), with frequent prompting (55%) and probing (48%). Sessions were mainly face-to-face (71%), audio recorded (64%), and transcribed verbatim. Thematic (38%) and content (26%) analyses predominated, often supported by NVivo. The Survey Response Model was the most cited theoretical framework (26%), though 22% of studies lacked any theoretical reference. Coding strategies were mixed (36%), and about half of the studies reported questionnaire revisions after analysis.

**Conclusions:**

TA remains a versatile but methodologically fragmented approach in questionnaire validation. The lack of standardization limits comparability and interpretability across studies. Greater transparency in reporting and explicit theoretical framing are recommended to improve methodological rigor.

**Registration:**

The review followed the PRISMA-ScR framework and was prospectively registered on OSF (DOI: https://doi.org/10.17605/OSF.IO/AZY3).

**Supplementary Information:**

The online version contains supplementary material available at https://doi.org/10.1186/s12874-026-02927-9.

## Background

### Rationale

Developing valid and reliable health questionnaires requires careful attention to how respondents understand and process survey questions. Cognitive interviewing has become a widely used method to identify problems of comprehension, interpretation, and response. This approach allows researchers to explore whether items are interpreted as intended and to detect potential sources of error in questionnaire design [1, 2].

One typology of cognitive interviews is thinking-aloud, a procedure during which the interviewee verbalizes his or her thoughts while reading a questionnaire [2]. When this method is applied, participants are asked to spontaneously report everything that goes through their minds while doing a task, and they are instructed not to interpret or analyse their thinking. The thinking-aloud methodology, as a stand-alone practice, was developed by Ericsson and Simon in 1984 and conceptualized as a means to uncover psychological mechanisms and knowledge structures underlying problem–solving activities (Ericsson and Simon, 1984). In their original framework, verbalizations were expected to remain close to the immediate thought process, without additional explanations or justifications, as these could trigger memory retrieval and distort the cognitive sequence. According to their theory, thinking-aloud could be performed both concurrently and retrospectively, a technique that foresees the interviewee recalls information only after having read the questionnaire [3]. However, the authors questioned the reliability of retrospective reports because of potential memory decay or post task rationalizations [4]. They also suggested that verbalization requests made by the interviewer could alter the way people think [5]. According to their theory, the analysis should be conducted by transcribing and segmenting utterances, then categorizing each segment without relying on the contextual aid of the preceding and following segment [6] .

The strict protocol proposed by the two authors was not fully supported on its methodological premises, as these theoretical assumptions were not broadly accepted within the psychological community and, for this reason, have been subject of debate concerning both interview conduction and data analysis. Critics argued that excluding contextual information from the analysis limited interpretability, and scholars such as Yang [6] advocated for a more comprehensive account of data collection, coding, and theoretical framing. Another challenge observed in practice was the participants often failed to verbalize their thoughts consistently, prompting researchers to adopt strategies such as pre-task orientation or prompting to encourage richer responses [5]. Yet, prompting itself was considered problematic by, some, as vocalizing while performing a task may feel unnatural and threaten validity. Retrospective thinking-aloud (RTA) protocols were proposed as an alternative, allowing participants to complete the task silently and comment performance afterwards [4, 7] Probing techniques, introduced in the 1980s, further enriched thinking-aloud interviews by eliciting additional details clarifying ambiguous utterances. Today, the combination of thinking-aloud and probing has become one of the most common approaches to cognitive interviewing [1].

Questionnaires are widely used in health research to measure perceptions, behaviours, and clinical outcomes, yet their validity depends on respondents correct interpretation of items, helping to detect sources of misinterpretation and improve instrument quality [8]. Even minor ambiguities can threaten measurement accuracy [9]. To address such challenges cognitive interview, which includes thinking-aloud and probing approaches, has been developed as a qualitative method to explore how individuals comprehend, retrieve, and formulate answers to survey questions. The World Health Organization, for instance, includes cognitive interviewing in the pre-testing phase of its Process of Translation and Adaptation of Instruments to ensure conceptual and cultural equivalence across versions [10]. Thinking-aloud techniques have been increasingly applied to assess how respondents interpret and answer questionnaire items, for example, to refine health surveys [11], patient-reported outcome measures [12, 13], and mental health screening tools [14], providing direct insight into comprehension and response processes and guiding instrument improvement.

However, despite decades of use, there is no gold standard for how thinking-aloud protocols should be designed, conducted, or analysed. Studies vary in sampling strategies, use of prompting and probing, analytic frameworks, and reporting practices [15]. This heterogeneity reduces comparability across studies and risks undermining the rigor of the approach [1].

### Objectives

The present scoping review aims to synthesize methodological approaches applied to the design and analysis of thinking-aloud protocols when applied to questionnaire development and validation. By examining concurrent, retrospective, and hybrid think-aloud approaches, together with prompting, probing, analytical strategies, and theoretical frameworks, this review seeks to characterize current methodological practices, clarify the state of the field, and identify priorities for future methodological research.

## Methods

This scoping review is reported according to the general methodology of the Preferred Reporting Items Extension for Scoping Review [16].

### Protocol and registration

The scoping review protocol was registered on the 16th of July 2025 with the following DOI: https://doi.org/10.17605/OSF.IO/AZY3, available at the link https://osf.io/azy3q/.

### Eligibility criteria

#### Inclusion criteria

We included original studies or research letter in English language that applied the thinking-aloud method for the development or validation of the health-related questionnaires. Within psychometric research, such validation through thinking-aloud has been differently defined, including:

- Assessing content validity and item intelligibility;
- testing comprehensibility, relevance, and answerability;
- evaluating usability, or.
- capturing respondents’ perceptions of questionnaire items.

#### Exclusion criteria

Studies were excluded if they:

1. didn’t actually used thinking aloud, or not used it to perform content validation;
2. applied thinking-aloud for usability testing of digital tools (apps, software, electronic records, educational resources, decision-making aids, self-management platforms, online support tools, manuals, vignettes);
3. used thinking-aloud to investigate general cognitive processes (e.g., decision making, problem solving, learning, writing, computational thinking, metacognition);
4. were the wrong type of publication: abstracts, book chapters, conference papers, or editorials. methodological papers;
5. involved domains outside health (e.g., teaching, engineering, chemistry, physics);
6. language other than English or Italian.

### Information sources

To identify potentially relevant documents, the search was conducted on the 17th of July 2025, without placing any time frame filter, on Pubmed, Scopus and Embase. This search query was formulated for PubMed and then applied with the necessary changes in Scopus and Embase. The final search results were exported into Covidence [1], where duplicates were automatically removed. The review was restricted to peer-reviewed publications because the objective was to examine and compare methodological approaches as formally reported in the scientific literature.

### Search

We used the following key terms, properly combined: *“Surveys and Questionnaires”*,* “Psychometrics” “Validation Studies as Topic” “Interviews as Topic”*, “valid*”,”verif*”, “questionnaire*”,”survey*”,”assess*”,” interview”*,“think* aloud”. The search strategy was developed by the review team with support from a researcher experienced in literature searching and evidence synthesis. The search string was subsequently reviewed by another member of the research team before implementation. All the search strategies are available in the Supplementary file 1.

### Selection of sources of evidence

Two reviewers (LT and FDO) independently screened titles and abstracts; disagreements were solved through discussion. The same two researchers screened the full texts, applying the pre-established exclusion criteria and solving disagreements by discussion.

#### Judgment of the comprehensiveness of description

Our review aim was to describe key features of how thinking-aloud process has been conducted in studies that stated the aim of validating a questionnaire; consequently, we were principally concerned with the comprehensiveness of the description of methods of applying the thinking-aloud approach, rather than appraising the quality of the study. Following Pollock [17], we defined as “complete” those studies that provided sufficient detail to permit replication of the procedure, following the essential elements delineated in the Cognitive Interview Reporting Framework [18]. One reviewer (LT) assessed all eligible studies, applying this criterion to determine whether the methodological description was complete or incomplete.

### Data charting process

A standardized data charting form was developed by two reviewers to guide extraction.

For all included articles, one reviewer (LT) recorded bibliographic information, research area, study aim, instrument tested, instrument development stage, and stated purpose of the thinking-aloud.

For the subset of studies judged “complete,” two reviewers (LT and MB) independently extracted further methodological details (e.g., type of thinking-aloud, use of prompting/probing, session characteristics, analysis method, theoretical framework). Discrepancies were resolved by discussion.

### Data items

#### Detailed data charting for the subset of complete studies

For studies judged to be “complete” the tabulated variables were:

##### Study and purpose

- Stated aim of the study (reduced to the following categories: developing and validating a questionnaire, translating, culturally adapting and validating a questionnaire, further validating an already finalized questionnaire, validating the content of a questionnaire, validating the construct, and testing the face validity).
- Stated purpose for applying the thinking-aloud method, reduced to the following categories: Content validation of the questionnaire, to test comprehension and clarity, for pilot and exploratory testing the questionnaire, for face validation, For testing acceptability and feasibility, for face and content validation, other.

##### Thinking-aloud procedure

- Typology of thinking-aloud protocol (concurrent, retrospective, hybrid).
- Use of prompting (yes/no) and type of prompting.
- Use of probing (yes/no) and type of probing (comprehension probes, evaluative probes, recall probes, concept coverage probes, acceptability/ feasibility probes, other probes and unspecified)).
- Prior instructions given to participants.
- Number of interview rounds.
- Average session duration.
- Round-specific revisions performed after interviews.

##### Sample and recruitment

- Sample type (patients, carers, users, mixed).
- Eligibility criteria.
- Recruitment strategy (purposive, saturation, pre-fixed).
- Rationale for sample size.

##### Data collection

- Mode of administration (in-person, telephone, online, mixed).
- Recording method (audio, video, audio with field notes).
- Transcription method (verbatim, selective).

##### Data analysis

- Analysis method (thematic analysis, content analysis, constant comparison methods, systematic structured approach, mixed or combined approach.).
- Coding type (inductive, deductive, mixed).
- Use of software (e.g., NVivo, MAXQDA, ATLAS.ti).
- Use of item tracking matrix.

##### Theoretical framework and outputs

- Theoretical or sociocultural framework (e.g., Survey Response Model, qualitative methodological models, guidelines, psychological/social theories).
- Reported outputs (e.g., questionnaire modification, cultural adaptation, theme identification, other insights).

Given the high heterogeneity of the approaches used to analysed data obtained applying the thinking- aloud method, we decided to operationalize the variable “Sociocultural/theoretical framing of data analysis as follow:

- Survey Response Models (includes Tourangeau in all its variables: four stage model, adapted, cognitive processing), Willis, Someren, Barnard and Sandberg, Conrad, Blair, Knafl et al., and general description as “cognitive interviewing theory” or “Survey response model” or “Analytical coding based on cognitive interviews”),
- Qualitative Methodological Models (includes Braun and Clarke, Miles and Huberman’s approach, Ritchie and Lewis, Kuckartz and Radiker, National centre for Sical Research Framework qualitative analysis method, Darker and French coding frame, Elo and Kyngas, Axial coding),
- Guidelines (COSMIN criteria, FDA guidelines o PRO development, EULAR and ISPOR guidelines),
- Psychological or Social Theories (Response expectancy theory (Kirsch), Common-Sense Model (Leventhal), Anxiety sensitivity theory, three-factor model underlying ASI-3, Mentalization theory, Gender and power theory (Connell), Social constructivism, gender schema theory, TTM theory, Construct validity theory and cognitive task analysis).

### Synthesis of results

To synthesize the evidence included in this scoping review, we adopted a narrative and tabular approach, aiming to address the research objectives of describing methodological practices in the use of thinking-aloud protocols for questionnaire development and validation. The synthesis first organizes studies according to their stated purpose (e.g., developing a new questionnaire, validating content or construct, translating and culturally adapting instruments), then describes key methodological characteristics across studies, including type of thinking-aloud (concurrent, retrospective, hybrid), use of prompting and probing, sample size and composition, session characteristics, transcription and recording methods, analysis approach, coding strategy, and theoretical or sociocultural framework.

Evidence is presented using summary tables that map the methodological choices to the study objectives, facilitating comparison across studies. Additional visual representations, such as heatmaps, illustrate the relationships between study purpose, analysis method, and theoretical framework. Descriptive statistics (e.g., frequency, median session duration, proportion of studies using prompting or probing) complement the narrative synthesis, providing a quantitative overview.

## Results

### Selection of sources of evidence

**Fig. 1:**
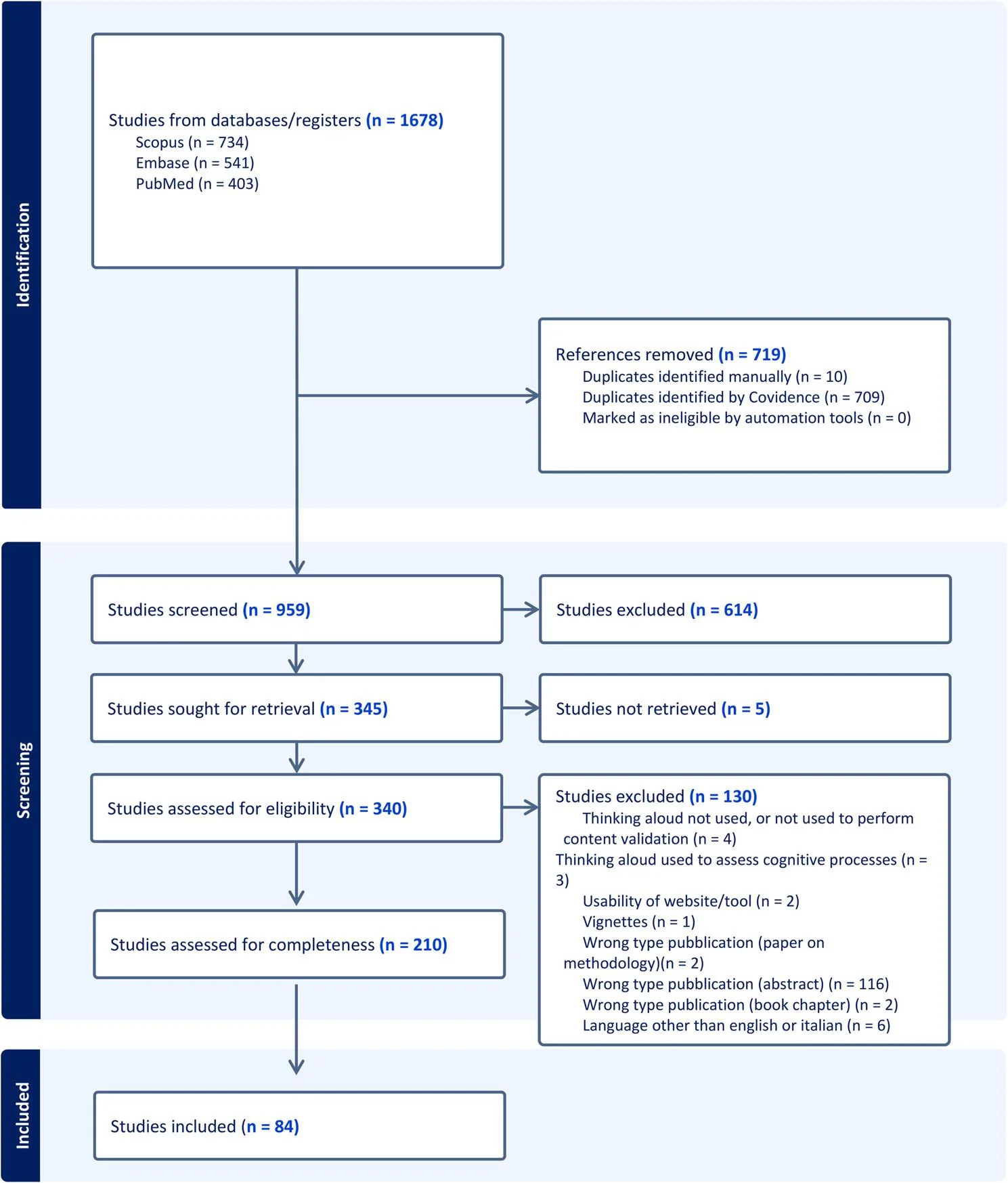
PRISMA Flowchart

A total of 1678 articles were retrieved from the searches, and these articles were reported into Covidence [19] for duplicate removal, with reduction of the total number of articles to 959 articles. Two researchers independently screened title and abstract, obtaining 345 eligible articles. Exclusion reasons of the eligible articles are summarized in the PRISMA flowchart (Fig. 1). At the end of the process, 210 articles were included for analysis. Of these, 84 were judged as complete in the description of the methodology.

### Characteristics of sources of evidence

Details of the 210 included articles are provided in the table of included studies (Supplementary file n.2).

As summarized in Table 1, the use of thinking-aloud methodologies in health questionnaire development and validation has increased substantially over time, with almost half of the included studies published between 2021 and 2025. Most studies were conducted within medicine and psychology and primarily focused on developing and validating new questionnaires or further validating existing instruments. The most frequently reported purpose of thinking-aloud was content validation, followed by the assessment of comprehension, clarity, acceptability, and feasibility.

**Table 1:** Characteristics of the 210 articles retrieved

	*n*.	%
Country		
Europe	81	38
Uk	48	23
USA	25	12
Australia	14	6
Canada	17	8
Asia	6	3
Africa	3	1
Others	16	8
Publication year		
2005–2010	13	6
2011–2015	34	16
2016–2020	64	30
2021–2025	99	47
Research area		
Medicine	119	56
Psychology	32	15
Nursing & Midewifery	18	8.5
Social sciences	12	5
Nutrition	9	4
Pharmacy	6	2
Other	14	7
Stated purpose of the included studies		
Developing and validating a questionnaire	79	37
Further validate an already finalized questionnaire	63	30
Translating, culturally adapting and validating a questionnaire	42	20
Validate the content	16	7
Validate the construct	4	1
Face validity	4	1
Other	2	0
Stated purpose of thinking-aloud		
Content validation of the questionnaire	80	37
To test comprehension and clarity	26	12
for pilot and exploratory testing the questionnaire	24	11
For face validation	22	10
For testing acceptability and feasibility	22	10
For face and content validation	13	6
Other	23	11

### Results of individual sources of evidences

The 84 articles judged to be “complete” in their description of the methodology used to analyse the thinking-aloud transcripts are summarized in Table 2 [20–103].

**Table 2:** Methodological features of included studies

Domain	Variable	Categories / Values	*n* (%) or Median
Sample characteristics	Sample type	Patients	41 (48%)
		Nonprofessional carers	5 (6%)
		Users	16 (19.0%)
		Mixed	7 (8%)
	Reason for stopping recruitment	Thematic saturation	32 (38%)
		Pre-fixed sample size	32 (38%)
		Purposive sampling	7 (8%)
		Unclear/not reported	13 (15%)
	Eligibility criteria clarified	Yes	76(90%)
		No / unclear	8 (9%)
Session characteristics	Thinking- Aloud approach	Concurrent	41 (48%)
		Retrospective	6 (7%)
		Hybrid	34 (40%)
	Prompting	Used	47 (56%)
	Probing	Used	41 (48%))
	Interviewer qualification clarified	Yes	32 (38.0%)
	Participant eligibility clarified	Yes	38 (45%)
Session context & data collection	Interview context	In person	60 (71%)
		Telephone	5 (5%)
		Online	11 (13%)
		Mixed (in person + remote)	2 (2%)
	Recording	Audio	54 (64%)
		Audio + field notes	18 (21%)
		Video	2 (2%)
	Transcription	Verbatim	80 (95%)
		Selective	4 (4%)
	Session duration	Median (minutes)	47.7
	Number of rounds	One	54 (64%)
		Two	15 (17%)
		Three or more	15 (17%)
Analysis methodologies	Analysis method	Thematic analysis	32 (38.0%)
		Content analysis	22 (26%)
		Constant comparative method	2 (2%)
		Framework analysis	2 (2%)
		Other / unspecified	6 (7%)
Theoretical / Sociocultural frameworks	Survey Response Model	Alone	22 (26%)
		With Qualitative methodological models	4 (4%)
		With Guidelines	3 (3%)
	Qualitative methodological models	Alone	9 (10%)
		With Psychological/Social theories	1 (1%)
		With Guidelines	3 (3%)
	Psychological/Social theories	Alone	7 (8%)
	Guidelines	Alone	16 (19%)
	Unspecified		19 (22%)
Coding and revision	Coding type	Deductive	25 (29%)
		Inductive	23 (27%)
		Mixed	31 (36%)
		Not mentioned/unclear	4 (4%)
	Item tracking matrix	Present	35 (41%)
	Round-specific revisions	Yes	33 (39%)
Software used	NVivo		24 (28%)
	ATLAS.ti		9 (17%)
	MAXQDA		5 (5%)
Output	Modification, deletion or addition of one or more questionnaire items		42 (50%)
	Cultural adaptation		27 (32%)
	Theme identification		70 (83%)

Patients were the most common sample category (48%), with a median sample size of 21 participants. Recruitment was usually discontinued for thematic saturation (38%) or based on predefined numbers (38%). Eligibility criteria were generally well defined (90%). Concurrent thinking-aloud protocols predominated (48%), and prompting (55%) and probing (48%) techniques were widely applied. Most sessions were face-to-face (71%), audio recorded (64%) and lasted a median of 47.7 min. Thematic (38%) and content (26%) analyses were the most frequently used. The Survey Response Model was the main theoretical framework (26%), though 22% of studies did not specify any one. Mixed coding strategies were slightly more prevalent (36%) than deductive (29%) or inductive (27%). NVivo was the most frequently used software for analysis (28%). 

### Synthesis of results

When analysing the theoretical framework adopted in the retrieved articles, for translation and cultural adaptation studies the Survey Response Model combined with content or thematic analysis was most frequently used. For questionnaire development, the most common combination was the use of guidelines as a framework paired with thematic analysis. Further validation studies typically applied guidelines and qualitative methodological models alongside thematic analysis. Construct validity studies consistently used psychological or social theories as frameworks, combined with thematic or content analysis. For content validation, survey response models and thematic analysis predominated. Face validation studies most often employed survey response models with either content or thematic analysis.

Several studies revised their questionnaires after analysing interview transcripts. Many modified, deleted, or added items, while others produced culturally adapted versions or identified central themes. Additional outcomes included changes to questionnaire instructions, insights into underreporting, guidance on whether questionnaires should be self- or proxy-completed, and the identification of issues encountered during completion. Appendix 3 presents a table in which the main methodological variations are summarised and their advantages and disadvantages discussed.

## Discussion

### Summary of evidence

In the process of developing a questionnaire, content validation plays a pivotal role, because it reflects the degree on which the instrument reflects the outcome it was intended to measure [8]. Content validation ensures that the questionnaire in comprehensive, relevant and understandable by the target audience, and it is particularly important when an instrument has to capture aspects related to the health aspects of individuals, especially when Patient reported Outcomes or Experiences are involved [104], because PROMS represent the patient’s point of view and give patient a voice during outcome evaluation [9]. A questionnaire has to be content validated also when translated in another language or applied to a population that differs from the one it was developed for.

Content validity can be performed in different, but always qualitative, ways: through Delphi studies, focus groups, cognitive interviews and opinion expert [8]. Thinking aloud is a form of cognitive interviewing, originating in the 1980s from cognitive psychology and increasingly used in the field of health sciences, specifically in PROMs. This is due to its relative ease of use, the minimal equipment required, and the opportunity it provides to gather direct feedback on the questionnaire from the target population [1].

The widespread adoption of thinking aloud has been however accompanied by considerable methodological variability. The lack of a standardized methodological framework has led to a diverse set of practices, which, while reflecting the flexibility of the approach, also raise questions about comparability and the interpretability of results.

The initial 210 studies retrieved span a broad temporal, disciplinary, and geographical spectrum, reflecting the widespread adoption of thinking-aloud methods. Most research was published in recent years across medicine and psychology journals, underscoring growing interdisciplinary interest in applying this approach to questionnaire development and validation.

Among the 84 studies providing complete methodological information, most involved patient samples and employed concurrent thinking-aloud protocols analysed through thematic or content analysis. Recruitment commonly ceased at thematic saturation or a predefined sample size, and sessions were typically face-to-face, audio recorded, and transcribed verbatim. The Survey Response Model emerged as the predominant theoretical framework, while coding approaches were evenly distributed across deductive, inductive, and mixed strategies. NVivo was the most frequently used software. Across studies, the use of thinking-aloud techniques primarily supported theme identification and questionnaire refinement, underscoring their role in enhancing instrument validity and cultural adequacy.

A first area of variability concerns sampling strategies and sample size. Although the median number of participants was around 20, studies differed considerably in their rationale for recruitment. This heterogeneity aligns with literature, given that some studies suggest that a small sample of five or six participants may already produce stable results [105], while seminal studies claim the need for larger numbers [106]. Caulton [107], pointed out that a heterogeneous sample of participants affects the relationship between sample size and stability and exhaustiveness of the problems detected. For this reason, studies employing mixed sample (for example, composed by patients and health care professionals) should aim for a larger sample compared with those recruiting only the target population of interest. In the retrieved studies, approximately half predetermined the sample size, while the others stopped recruitment upon reaching saturation, reflecting an ongoing debate in qualitative research: whether to pursue complete, time-consuming, and potentially endless thematic saturation, or to adopt a more pragmatic, but likely less exhaustive, pre-defined sample size [108].

Regarding thinking-aloud procedures, concurrent and hybrid protocols predominated, while retrospective approaches were less frequently employed. The choice between the concurrent and retrospective methods depends largely on the author’s main objective: minimizing thought alteration and the potential reactivity associated with the concurrent approach or obtaining a richer but more rationalized account through retrospective reporting [109] The frequent use of prompting and probing, as well as the use of the hybrid approach, suggests an outcome-oriented stance. Many researchers often found that participants struggled to verbalize spontaneously, reporting earlier concerns about reactivity and the artificiality of the method [2]. Probing helps retrieving information not spontaneously provided during the interview. Yet, the inclusion of these techniques introduces potential interviewer effects, raising concerns about validity. The use of prompting and probing, even if with the risk of introducing researcher’s bias, seem to be a reliable method to obtain information even from those subjects that are not able, or are not inclined, to verbalise their thoughts while reading. The use of these techniques is suggested tough to maximize results from interviews.

The analysis of transcripts was mainly performed through thematic and content analysis, often supported by qualitative software, but with a wide variation on the application of inductive, deductive, and mixed coding approaches. This suggests that researchers tend to tailor their analytic strategies to the purpose of their study, or to the technique with which they are most familiar, rather than adhering to a shared methodological standard. Importantly, the Survey Response Model emerged as the most cited theoretical framework, but a considerable number of studies failed to specify any explicit framework, failing on one of the methodological step considered essential by ISPOR guidelines when developing a measurement instrument [104]. Such omission limits transparency and weakens the interpretative grounding of the findings, reinforcing Yang’s (2003) call for explicit reporting of theoretical underpinnings. This is even more true if we consider that for Eriksonn and Simon [110] data obtained from thinking-aloud should have been used to validate a pre-existent theoretical model and cannot be interpreted alone [111]. In qualitative research, adopting a theoretical framework helps provide the conceptual hierarchy when developing the questionnaire [112], and then to give a sense of coherence and a narrative sense to the themes emerging from the data, allowing insights to extend beyond participants’ literal words [113]. It is plausible that when the thinking-aloud method is transferred from psychology to medicine, it loses much of its theoretical foundation and retains primarily its pragmatic dimension, relying mainly on the literal interpretation of transcripts [114].

In terms of outputs, thinking-aloud protocols consistently informed questionnaire revisions, ranging from item modification to cultural adaptations. On this topic, a substantial methodological issue concerns the numbers of rounds of interview performed to validate the questionnaire. As suggested also in ISPOR [104] and COSMIN guidelines [8], when a questionnaire is revised following a round of cognitive interviews, it should ideally be re-tested with a different sample in order to confirm the validity of modifications. At the same time, it is important to recognize that addressing every concern or suggestion raised during interviews may not always be appropriate or necessary.

A still unexplored but promising area is the use of artificial intelligence in the segmentation and coding of interviews transcripts for questionnaire validation. Borse’s work demonstrated a satisfactory inter-rater reliability between researcher and GPT 4.5 analysis of an audio transcript, suggesting the possibility of overcoming the time-consuming limitation of analysing a large body of transcripts. As in other fields, achieving reliable results is strongly dependent on appropriate prompt design [115].

Taken together, these findings indicate that thinking-aloud method remains a powerful yet methodologically fragmented tool. Nevertheless, the diversity of approaches observed— in data collection, analysis, or theoretical framing—suggests that results across studies may not be directly comparable. Future research should move toward the confrontation of different approaches on the validation of the same instrument, as done in Alshammari’s work, where a confrontation between concurrent and retrospective thinking-aloud approach in a usability test was performed [7]. Only through such efforts the superiority, or the advantages and the disadvantages of a methodology, can be stated, and clear indications produced, other than simple suggestions based on a pragmatic approach, the same that are used for the formulation of the guidelines cited along the article.

### Limitations

Although PubMed, Embase, and Scopus provide broad coverage of the biomedical, health sciences, and interdisciplinary literature, studies indexed exclusively in other databases may have been missed. Furthermore, although all screening and study selection procedures were conducted independently by two reviewers, the assessment of methodological completeness was performed by a single reviewer and may therefore be subject to subjective interpretation. As a result, some studies may have been classified differently, potentially influencing the characterization of methodological practices reported in this review.

## Conclusions

This scoping review shows that thinking-aloud methodologies are widely used in health questionnaire development and validation, but are characterized by substantial heterogeneity in their design, conduct, analysis, and reporting. While this flexibility may facilitate adaptation to different research contexts, it also limits the comparability and reproducibility of findings across studies.

Greater transparency in reporting methodological choices, theoretical frameworks, analytical procedures, and questionnaire revisions would strengthen the rigor of future studies. Further methodological research should directly compare alternative thinking-aloud approaches, including concurrent, retrospective, and hybrid protocols, to better understand their respective strengths and limitations and support more consistent practices in questionnaire validation. 

## Supplementary Information

Supplementary Material 1.

Supplementary Material 2.

Supplementary Material 3.

## Data Availability

Data are available on request to the authors.

## References

[1] Willis GB, Artino AR. What Do Our Respondents Think We’re Asking? Using Cognitive Interviewing to Improve Medical Education Surveys. J Grad Med Educ. 2013;5(3):353–6. 10.4300/jgme-d-13-00154.1 . PubMed PMID: 24404294. PMC3771159 24404294

[2] Priede C, Farrall S. Comparing results from different styles of cognitive interviewing: verbal probing vs. thinking aloud. Int J Soc Res Methodol. 2011;14(4):271–87. 10.1080/13645579.2010.523187.

[3] Fonteyn ME, Kuipers Benjamin, Grobe Susan J. A description of Think Aloud Method and Protocol Analysis. Qual Health Res. 1993;3(4):430–331.

[4] Alhadreti O, Mayhew P. Rethinking thinking aloud: A comparison of three think-aloud protocols. In: Conference on Human Factors in Computing Systems - Proceedings. Association for Computing Machinery; 2018. 10.1145/3173574.3173618.

[5] Charters E. The Use of Think-aloud Methods in Qualitative Research An Introduction to Think-aloud Methods. Brock education journal. 2003;12(2). 10.26522/brocked.v12i2.38.

[6] Ching Yang S. Reconceptualizing think-aloud methodology: refining the encoding and categorizing techniques via contextualized perspectives. Comput Hum Behav. 2003;19(1):95–115. 10.1016/S0747-5632(02)00011-0.

[7] Alshammari T, Alhadreti O, Mayhew PJ. When to Ask Participants to Think Aloud: A Comparative Study of Concurrent and Retrospective Think-Aloud Methods. International Journal of Human Computer Interaction (IJHCI). 2015. Available from: https://www.ueaticketbookings.co.uk.

[8] Mokkink LB, Herbelet S, Tuinman PR, Terwee CB. Content validity: judging the relevance, comprehensiveness, and comprehensibility of an outcome measurement instrument – a COSMIN perspective. J Clin Epidemiol. 2025;185. 10.1016/j.jclinepi.2025.111879 . PubMed PMID: 40562250. 40562250

[9] MacDermid JC. ICF Linking and Cognitive Interviewing Are Complementary Methods for Optimizing Content Validity of Outcome Measures: An Integrated Methods Review. Front Rehabilitation Sci Front Media SA. 2021. 10.3389/fresc.2021.702596. PMC9397968 36188847

[10] World Health Organization. Process of translation and adaptation of instruments. Geneva: WHO; 2016. https://www.who.int/substance_abuse/research_tools/translation/en/.

[11] Monaca C, Weigl M, Pfaff H, Hammer A. Safety Culture Through Patient Voice: Qualitative Validation of the Patients’ Perceptions of Safety Culture Scale (PaPSC) in Cardiology and Cardiothoracic Surgery. Health Expect. 2025;28(2). 10.1111/hex.70213 . PubMed PMID: 40045553. PMC11882746 40045553

[12] Balstad TR, Bye A, Jenssen CRS, Solheim TS, Thoresen L, Sand K. Patient interpretation of the patient-generated subjective global assessment (PG-SGA) short form. Patient Prefer Adherence. 2019;13:1391–400. 10.2147/PPA.S204188. PMC6701615 31496666

[13] Bentley S, Findley A, Chiva-Razavi S, Naujoks C, Patalano F, Johnson C, et al. Evaluation of the content validity of patient-reported outcome (PRO) instruments developed for use with individuals with phakic presbyopia, including the Near Activity Visual Questionnaire-presbyopia (NAVQ-P) and the near vision correction independence (NVCI) instrument. J Patient Rep Outcomes. 2021;5(1). 10.1186/s41687-021-00379-x . PubMed PMID: 34689253. PMC8542063 34689253

[14] Brown GP, Hawkes NC, Tata P. Construct validity and vulnerability to anxiety: A cognitive interviewing study of the revised Anxiety Sensitivity Index. J Anxiety Disord. 2009;23(7):942–9. 06.001 PubMed PMID: 19586752. 10.1016/j.janxdis.2009.06.001 19586752

[15] Koro-Ljungberg M, Douglas EP, Therriault D, Malcolm Z, McNeill N. Reconceptualizing and decentering think-aloud methodology in qualitative research. Qualitative Res. 2013;13(6):735–53. 10.1177/1468794112455040.

[16] Tricco AC, Lillie E, Zarin W, O’Brien KK, Colquhoun H, Levac D, et al. PRISMA Extension for Scoping Reviews (PRISMA-ScR): Checklist and Explanation. Ann Intern Med. 2018;169(7):467–73. 10.7326/M18-0850. 30178033

[17] Pollock A, Campbell P, Struthers C, Synnot A, Nunn J, Hill S, et al. Stakeholder involvement in systematic reviews: A protocol for a systematic review of methods, outcomes and effects. Res Involv Engagem. 2017;3(1). 10.1186/s40900-017-0060-4. PMC5611627 29062534

[18] Willis G, Boeije H, editors. Joop Hox and Nekane Balluerka Special Issue Cognitive Interviewing Reporting Framework Guest Editors Offi cial Organ of the European Association of Methodology 3 / 13 Contents. Available from: https://www.hogrefe.com/journals/methodology

[19] Covidence systematic review software. Melbourne, Australia: Veritas Health Innovation. https://www.covidence.org.

[20] Shedden-Mora M, Alberts J, Petrie K, Laferton J, Nestoriuc Y, Lowe B. The treatment expectation questionnaire (TEX-Q)- A generic multidimensional scale measuring patients’/INS; treatment expectations. J Psychosom Res. 2019;Seventh Annual Scientific Conference of the European Association of Psychosomatic Medicine (EAPM). Rotterdam Netherlands.121:129–30. 10.1016/j.jpsychores.2019.03.090 PubMed PMID: 2001860508.

[21] Despina A, Stylianos K, Irene P, Elisabeth P, Aliki T. Translation and cultural adaptation of the Greek integrated palliative care outcome scale (IPOS): challenges in a six-phase process. BMC Palliat Care. 2023;22(1). 10.1186/s12904-023-01278-2 . PubMed PMID: 37919682. PMC10621102 37919682

[22] Andersen RD, Jylli L, Ambuel B. Cultural adaptation of patient and observational outcome measures: a methodological example using the COMFORT behavioral rating scale. Int J Nurs Stud. 2014;51(6):934–42. 10.1016/j.ijnurstu.2013.10.006 . PubMed PMID: 609123832. 24182731

[23] Bailey C, Tully M, Cooke J. An investigation into the content validity of the Antimicrobial Self-Assessment Toolkit for NHS Trusts (ASAT v15a) using cognitive interviews with antimicrobial pharmacists. J Clin Pharm Ther. 2015;40(2):208–12. 10.1111/jcpt.12248 . PubMed PMID: 602331493. 25678341

[24] Balstad TR, Bye A, Jenssen CRS, Solheim TS, Thoresen L, Sand K. Patient interpretation of the patient-generated subjective global assessment (PG-SGA) short form. Patient Prefer Adherence. 2019;13:1391–400. 10.2147/PPA.S204188. PMC6701615 31496666

[25] Tatlock S, Bentley S, Arbuckle R, Abetz-Webb L, Scott J. Content validation of a caregiver diary to monitor severity and recovery of pediatric patients with respiratory syncytial virus infection. J Patient Rep Outcomes. 2022;6(1):48. 10.1186/s41687-022-00442-1 . PubMed PMID: 645113018. PMC9098775 35554751

[26] Blackmore AM, Mulhern B, Norman R, Reddihough D, Choong CS, Jacoby P, et al. How Well Does the EQ-5D-Y-5L Describe Children With Intellectual Disability? There’s a Lot More to My Child Than That She Can’t Wash or Dress Herself. Value Health. 2024;27(2):190–8. 10.1016/j.jval.2023.11.005 . PubMed PMID: 2029279187. 38043713

[27] Brod M, Waldman LT, Shu AD, Smith A. Content validation of the SF-36v2 Health Survey Acute for use in hypoparathyroidism. Qual Life Res. 2023;32(6):1795–806. 10.1007/s11136-023-03352-x . PubMed PMID: 2021403115. PMC10172226 36759379

[28] Broderick L, Foster A, Waldman LT, Jacobs KD, Bordone L, Yarlas A. Content validation of the Angioedema Quality of Life Questionnaire (AE-QoL) in a population of adult and adolescent patients with hereditary angioedema (HAE). J Patient Rep Outcomes. 2025;9(1). 10.1186/s41687-025-00876-3 . PubMed PMID: 40220084. PMC11993509 40220084

[29] Brown GP, Hawkes NC, Tata P. Construct validity and vulnerability to anxiety: A cognitive interviewing study of the revised Anxiety Sensitivity Index. J Anxiety Disord. 2009;23(7):942–9. 10.1016/j.janxdis.2009.06.001 . PubMed PMID: 50571749. 19586752

[30] Byrne R, Terranova CO, Chai LK, Brookes DSK, Trost SG. Cognitive Testing of Items Measuring Movement Behaviours in Young Children Aged Zero to Five Years: Development of the Movement Behaviour Questionnaires for -Baby (MBQ-B) and -Child (MBQ-C). Children. 2023;10(9). 10.3390/children10091554. PMC10529515 37761515

[31] Rand S, Caiels J, Collins G, Forder J. Developing a proxy version of the Adult social care outcome toolkit (ASCOT). Health Qual Life Outcomes. 2017;15(1):108. 10.1186/s12955-017-0682-0 . PubMed PMID: 616283535. PMC5438504 28526055

[32] Closson K, Zharima C, Kuchena M, Dietrich JJ, Gadermann A, Ogilvie G, et al. I feel like it is asking if he is a stalker … but I also feel like it is asking if he cares: exploring young South African women and men’s perceptions of the Sexual Relationship Power Scale. BMC Public Health. 2022;22(1):1368. 10.1186/s12889-022-13686-9 . PubMed PMID: 638504531. PMC9288208 35842627

[33] Coats H, Rosenfeld AG, Crist JD, Sternberg E, Berger A. Using cognitive interviews to improve a Psychological-Social-Spiritual Healing instrument: Voices of aging African Americans with serious illness. Appl Nurs Res. 2018;39:109–14. 10.1016/j.apnr.2017.11.019 . PubMed PMID: 626212413. PMC5808604 29422143

[34] Coleman S, Nixon J, Keen J, Muir D, Wilson L, McGinnis E, et al. Using cognitive pre-testing methods in the development of a new evidenced-based pressure ulcer risk assessment instrument. BMC Med Res Methodol. 2016;16(1):158. PubMed PMID: 618906022. 10.1186/s12874-016-0257-5 PMC5112672 27852237

[35] Coombes L, Braybrook D, Scott H, Harardottir D, Waite F, Ellis-Smith C et al. Comprehensibility, Comprehensiveness and Acceptability of a Novel Paediatric Palliative Care Outcome Measure: A Cognitive Interview Study with Children and Families. Palliat Med. 2022;12th World Research Congress of the European Association for Palliative Care, EAPC 2022. Online.36(1 SUPPL):34. 10.1177/02692163221093145 PubMed PMID: 638325932.

[36] de la Dornonville FL, Norup A, Schow T, Andersen TE. Evaluation of Response Processes to the Danish Version of the Dutch Multifactor Fatigue Scale in Stroke Using the Three-Step Test-Interview. Front Hum Neurosci. 2021;15. 10.3389/fnhum.2021.642680. PMC8134536 34025374

[37] Drakenberg A, Sluys KP, Ericsson E, Sundqvist AS. The Family Involvement in Care Questionnaire —An instrument measuring family involvement in inpatient care. PLoS ONE. 2023;18(8 August). 10.1371/journal.pone.0285562. PMC10426968 37582093

[38] Drolet-Labelle V, Bedard A, Lemieux S, Drapeau V, Vanderlee L, Laurin D, et al. Development and cognitive testing of a food-frequency questionnaire to assess intake of plant-based protein foods among older adults. Public Health Nutr. 2024;28(1):e16. 10.1017/S1368980024002052 . PubMed PMID: 645569299. PMC11822641 39431365

[39] Eardley S, Bishop FL, Cardini F, Santos-Rey K, Jong MC, Ursoniu S, et al. A pilot feasibility study of a questionnaire to determine European Union-wide CAM use. Forsch Komplementmed. 2012;19(6):302–10. PubMed PMID: 563039535. 10.1159/000345839 23343585

[40] Edwards M, Thomsen SC, Toroitich-Ruto C. Thinking Aloud to Create Better Condom Use Questions. Field methods. 2005;17(2):183–99. 10.1177/1525822X04271029.

[41] Egger-Rainer A. Enhancing validity through cognitive interviewing. A methodological example using the Epilepsy Monitoring Unit Comfort Questionnaire. J Adv Nurs. 2019;75(1):224–33. 10.1111/jan.13867 . PubMed PMID: 625597891. PMC7379296 30289559

[42] Eland ND, Strand LI, Ostelo RW, Kvale A, Magnussen LH. How do physiotherapists understand and interpret the Pain Attitudes and Beliefs Scale? A cognitive interview study. Physiother Theory Pract. 2022;38(4):513–27. 10.1080/09593985.2020.1774949 . PubMed PMID: 632046736. 32520655

[43] Engel L, Kosowicz L, Bogatyreva E, Batchelor F, Devlin N, Dow B, et al. Face Validity of Four Preference-Weighted Quality-of-Life Measures in Residential Aged Care: A Think-Aloud Study. Patient. 2023;16(6):655–66. 10.1007/s40271-023-00647-6 . PubMed PMID: 2025923326. PMC10570159 37803217

[44] Evans M, Gregory A, Feder G, Howarth E, Hegarty K. Even ‘daily’ is not enough: How well do we measure domestic violence and abuse?-A think-aloud study of a commonly used self-report scale. Violence Vict. 2016;31(1):3–26. 10.1891/0886-6708.VV-D-15-00024. 26645540

[45] Farmer N, Powell-Wiley TM, Middleton KR, Brooks AT, Mitchell V, Troncoso M, et al. Use of a focus group-based cognitive interview methodology to validate a cooking behavior survey among African-American adults. Front Nutr. 2022;9. 10.3389/fnut.2022.1000258. PMC9760831 36545469

[46] Bentley S, Findley A, Chiva-Razavi S, Naujoks C, Patalano F, Johnson C, et al. Evaluation of the content validity of patient-reported outcome (PRO) instruments developed for use with individuals with phakic presbyopia, including the Near Activity Visual Questionnaire-presbyopia (NAVQ-P) and the near vision correction independence (NVCI) instrument. J Patient Rep Outcomes. 2021;5(1):109. 10.1186/s41687-021-00379-x . PubMed PMID: 643921379. PMC8542063 34689253

[47] Gabes M, Ragamin A, Baker A, Kann G, Donhauser T, Gabes D, et al. Content validity of the Recap of atopic eczema (RECAP) instrument in Dutch, English and German to measure eczema control in young people with atopic eczema: a cognitive interview study. Br J Dermatol. 2022;187(6):919–26. 10.1111/bjd.21767 . PubMed PMID: 2018709185. 35842231

[48] Gadermann AM, Guhn M, Zumbo BD. Investigating the Substantive Aspect of Construct Validity for the Satisfaction with Life Scale Adapted for Children: A Focus on Cognitive Processes. Soc Indic Res. 2011;100(1):37–60. 10.1007/s11205-010-9603-x.

[49] Garfield K, Husbands S, Thorn JC, Noble S, Hollingworth W. Development of a brief, generic, modular resource-use measure (ModRUM): cognitive interviews with patients. BMC Health Serv Res. 2021;21(1):371. 10.1186/s12913-021-06364-w . PubMed PMID: 634863230. PMC8058988 33882905

[50] Gerlach C, Taylor K, Ferner M, Munder M, Weber M, Ramsenthaler C. Challenges in the cultural adaptation of the German Myeloma Patient Outcome Scale (MyPOS): An outcome measure to support routine symptom assessment in myeloma care. BMC Cancer. 2020;20(1):245. 10.1186/s12885-020-06730-7 . PubMed PMID: 631269387. PMC7092563 32293347

[51] Giles SJ, Lawton RJ, Din I, McEachan RRC. Developing a patient measure of safety (PMOS). BMJ Qual Saf. 2013;22(7):554–62. 10.1136/bmjqs-2012-000843 . PubMed PMID: 52474348. 23447658

[52] Hancox JE, Quested E, Duda JL. Suitability of the Perceived Motivational Climate in Sport Questionnaire-2 for Dance Research: A Think Aloud Approach. J Dance Med Sci. 2015;19(4):149–62. 10.12678/1089-313X. 19.4.149 PubMed PMID: 612573227. 26641702

[53] Horwood J, Sutton E, Coast J. Evaluating the Face Validity of the ICECAP-O Capabilities Measure: A Think Aloud Study with Hip and Knee Arthroplasty Patients. Appl Res Qual Life. 2014;9(3):667–82. 10.1007/s11482-013-9264-4.

[54] Humphrey L, Kulich K, Deschaseaux C, Blackburn S, Maguire L, Stromberg A. The Caregiver Burden Questionnaire for Heart Failure (CBQ-HF): Face and content validity. Health Qual Life Outcomes. 2013;11(1):84. 10.1186/1477-7525-11-84 . PubMed PMID: 52601969. PMC3673843 23706131

[55] Iqbal MZ, Ivan R, Robb C, Derby J. Evaluating factors that impact scoring an open response situational judgment test: a mixed methods approach. Front Med (Lausanne). 2024;11:1525156. 10.3389/fmed.2024.1525156 . PubMed PMID: 2032938351. PMC11743161 39835093

[56] Jeffrey NK, Senn CY. Gender Differences in Sexual Violence Victimization Experiences and Validity of Victimization Reports: A Think-Aloud Study. J Sex Res. 2024;1–17. 10.1080/00224499.2024.2397496 . PubMed PMID: 645242793. 39250419

[57] Kaklamanou D, Armitage CJ, Jones CR. A further look into compensatory health beliefs: a think aloud study. Br J Health Psychol. 2013;18(1):139–54. PubMed PMID: 369254847. 10.1111/j.2044-8287.2012.02097.x 23006056

[58] Kaltenbrunner M, Bengtsson L, Mathiassen SE, Engstrom M. A questionnaire measuring staff perceptions of Lean adoption in healthcare: development and psychometric testing. BMC Health Serv Res. 2017;17(1):235. 10.1186/s12913-017-2163-x . PubMed PMID: 618154319. PMC5364711 28340573

[59] Kaltenbrunner M, Hagerman H, Fagerstrom C, Hartveit M, Nordheim E, Ekstedt M. The Implementation Process Assessment Tool: translation, contextualization, and psychometric evaluation of a Swedish version in a municipal elderly care context. BMC Health Serv Res. 2024;24(1):1391. 10.1186/s12913-024-11889-. x PubMed PMID: 645770977. PMC11558869 39533292

[60] Kassam A, Douglas ML, Simon J, Cunningham S, Fassbender K, Shaw M, et al. Behaviors in Advance Care Planning and ACtions Survey (BACPACS): Development and validation part 1. BMC Palliat Care. 2017;16(1):56. 10.1186/s12904-017-0236-6 . PubMed PMID: 619343381. PMC5700663 29166904

[61] Khanna D, Lay K, Khadka J, Mpundu-Kaambwa C, Ratcliffe J. How do children understand and respond to the EQ-5D-Y-3L? A mixed methods study in a community-based sample of 6-12-year-olds. Health Qual Life Outcomes. 2024;22(1):105. 10.1186/s12955-024-02320-4 . PubMed PMID: 2032514256. PMC11619400 39633400

[62] Krieger B, Ederer F, Amann R, Morgenthaler T, Schulze C, Dawal B. Translation and cross-cultural adaptation of the young children participation and environment measure for its use in Austria, Germany, and Switzerland. Front Pediatr. 2023;11:1258377. 10.3389/fped.2023.1258377 . PubMed PMID: 2027945774. PMC10794623 38239598

[63] Krishna-Naik V, Palmer A, Hodson NA, Tugnait A, O’Connor DB. Utilisation of a think-aloud protocol to validate a self-reported periodontitis questionnaire. J Dent. 2024;150:105381. 10.1016/j.jdent.2024.105381 . PubMed PMID: 645424719. 39349094

[64] Lay K, Crocker M, Engel L, Ratcliffe J, Milte R, Hutchinson C. Exploring older people’s understanding of the QOL-ACC, a new preference-based quality-of-life measure, for quality assessment and economic evaluation in aged care: the impact of cognitive impairment and dementia. Health Qual Life Outcomes. 2024;22(1):4. 10.1186/s12955-023-02222-x . PubMed PMID: 2027787658. PMC10773014 38185654

[65] Manns S, Cramp F, Lewis R, Clark EM, Palmer S. A qualitative evaluation of the appropriateness, validity, acceptability, feasibility and interpretability of the Bristol Impact of Hypermobility (BIoH) questionnaire. Musculoskelet Sci Pract. 2018;38:69–76. 10.1016/j.msksp.2018.10.002 . PubMed PMID: 30312837. 30312837

[66] Martin S, Shah SN, Hepp Z, Harris N, Morgans AK. Qualitative Analysis of Pain in Patients With Locally Advanced or Metastatic Urothelial Carcinoma. Bladder Cancer. 2022;8(1):45–53. 10.3233/BLC-211518 . PubMed PMID: 637616319. PMC11181679 38994514

[67] McClam M, Workman L, Dias EM, Walker TJ, Brandt HM, Craig DW, et al. Using cognitive interviews to improve a measure of organizational readiness for implementation. BMC Health Serv Res. 2023;23(1):93. 10.1186/s12913-022-09005-y . PubMed PMID: 640143094. PMC9881511 36707829

[68] McCorry NK, Scullion L, McMurray CM, Houghton R, Dempster M. Content validity of the illness perceptions questionnaire - revised among people with type 2 diabetes: A think-aloud study. Psychol Health. 2013;28(6):675–85. 10.1080/08870446.2012.746690 . PubMed PMID: 52364698. 23252620

[69] McLoughlin C, Goranitis I, Al-Janabi H. The Feasibility and Validity of Preference-Based Quality of Life Measures With Informal Carers: A Think-Aloud Study. Value Health. 2023;26(11):1655–64. 10.1016/j.jval.2023.07.002 . PubMed PMID: 2026533980. 37516197

[70] Mertens VC, Moser A, Verbunt J, Smeets R, Goossens M. Content Validity of the Credibility and Expectancy Questionnaire in a Pain Rehabilitation Setting. Pain Pract. 2017;17(7):902–13. 10.1111/papr.12543 . PubMed PMID: 27911035. 27911035

[71] Mitchell PM, Caskey FJ, Scott J, Sanghera S, Coast J. Response process validity of three patient reported outcome measures for people requiring kidney care: A think-aloud study using the EQ-5D-5L, ICECAP-A and ICECAP-O. BMJ Open. 2020;10(5):e034569. 10.1136/bmjopen-2019-034569 . PubMed PMID: 631779716. PMC7232621 32414822

[72] Monaca C, Weigl M, Pfaff H, Hammer A. Safety Culture Through Patient Voice: Qualitative Validation of the Patients’ Perceptions of Safety Culture Scale (PaPSC) in Cardiology and Cardiothoracic Surgery. Health Expect. 2025;28(2):e70213. 10.1111/hex.70213 . PubMed PMID: 646738844. PMC11882746 40045553

[73] Namisango E, Luyirika EBK, Berger A. Cross Cultural Adaptation and Cognitive Testing of a Psycho-Social-Spiritual Healing Measure, the NIH Healing Experiences in All Life Stressors-NIH-HEALS. Glob Adv Health Med. 2022;11. 10.1177/21649561211067189. PMC8793392 35096489

[74] Nikolaus CJ, Ellison B, Nickols-Richardson SM. College students’ interpretations of food security questions: results from cognitive interviews. BMC Public Health. 2019;19(1):1282. 10.1186/s12889-019-7629-9 . PubMed PMID: 629575009. PMC6788030 31604466

[75] Olsson E, Anderzen-Carlsson A, Atladottir SM, Axelin A, Campbell-Yeo M, Eriksson M, et al. Cultural adaptation and harmonization of four Nordic translations of the revised Premature Infant Pain Profile (PIPP-R). BMC Pediatr. 2018;18(1):349. 10.1186/s12887-018-1322-5 . PubMed PMID: 624778057. PMC6225673 30409118

[76] Pandina G, Ness S, Trudeau J, Stringer S, Knoble N, Lenderking WR, et al. Qualitative evaluation of the Autism Behavior Inventory: use of cognitive interviewing to establish validity of a caregiver report scale for autism spectrum disorder. Health Qual Life Outcomes. 2021;19(1):26. 10.1186/s12955-020-01665-. w PubMed PMID: 2010213241. PMC7819236 33472654

[77] Park E, Kwon M. Testing the digital health literacy instrument for adolescents: Cognitive interviews. J Med Internet Res. 2021;23(3):e17856. 10.2196/17856 . PubMed PMID: 2011598182. PMC8074835 33720031

[78] Paterson H, Hay-Smith J, Treharne G, Herbison P, Howarth C. Validation of the Intuitive Eating Scale in pregnancy. J Health Psychol. 2018;23(5):701–9. 10.1177/1359105316671186 . PubMed PMID: 627154919. 28810354

[79] Penton H, Dayson C, Hulme C, Young T. A Qualitative Investigation of Older Adults’ Conceptualization of Quality of Life and a Think-Aloud Content Validation of the EQ-5D-5L, SF-12v2, Warwick Edinburgh Mental Wellbeing Scale, and Office of National Statistics-4. Value Health. 2022;25(12):2017–27. 10.1016/j.jval.2022.04.1735 . PubMed PMID: 35760713. 35760713

[80] Plazar D, Metyovinyi Z, Medvecz M, Rencz F. Qualitative evidence on EQ-5D-5L skin irritation and self-confidence bolt-ons in Darier’s disease and Hailey-Hailey disease. Qual Life Res. 2024. 10.1007/s11136-024-03871-1 . PubMed PMID: 2032686612. PMC11982107 39704914

[81] Pool JJM, Hiralal SR, Ostelo RWJG, van der Veer K, de Vet HCW. Added value of qualitative studies in the development of health related patient reported outcomes such as the pain coping and cognition list in patients with sub-acute neck pain. Man Ther. 2010;15(1):43–7. 10.1016/j.math.2009.05.010 . PubMed PMID: 19632876. 19632876

[82] Rand S, Caiels J, Collins G, Forder J. Developing a proxy version of the Adult social care outcome toolkit (ASCOT). Health Qual Life Outcomes. 2017;15(1). 10.1186/s12955-017-0682-0 . PubMed PMID: 28526055. PMC5438504 28526055

[83] Renberg T, Kettis Lindblad Å, Tully MP. Testing the validity of a translated pharmaceutical therapy-related quality of life instrument, using qualitative think aloud methodology. J Clin Pharm Ther. 2008;33(3):279–87. 10.1111/j.1365-2710.2008.00921. .x PubMed PMID: 18452415. 18452415

[84] Rencz F, Mukuria C, Bató A, Poór AK, Finch AP. A qualitative investigation of the relevance of skin irritation and self-confidence bolt-ons and their conceptual overlap with the EQ-5D in patients with psoriasis. Qual Life Res. 2022;31(10):3049–60. 10.1007/s11136-022-03141. -y PubMed PMID: 35471487. PMC9039271 35471487

[85] Sousa deR. Linguistic and cultural adaptation of the EARP Questionnaire to European Portuguese. Available from: https://www.arprheumatology.com. 36617309

[86] Rohr M, Brandstetter S, Plomer AS, Loss J, Kretschmer R, Apfelbacher C. A qualitative study exploring content validity and feasibility of frequently used generic health-related quality of life measures in older people with hip fracture: The patients’ perspective. Injury. 2021;52(2):134–41. 10.1016/j.injury. .2020.09.061 PubMed PMID: 33039180. 33039180

[87] Saldaris J, Weisenberg J, Pestana-Knight E, Marsh ED, Suter B, Rajaraman R, et al. Content Validation of Clinician-Reported Items for a Severity Measure for CDKL5 Deficiency Disorder. J Child Neurol. 2021;36(11):998–1006. doi:10.1177/08830738211019576 PubMed PMID: 34378447. 10.1177/08830738211019576 PMC8458223 34378447

[88] Schildmann EK, Groeneveld EI, Denzel J, Brown A, Bernhardt F, Bailey K, et al. Discovering the hidden benefits of cognitive interviewing in two languages: The first phase of a validation study of the Integrated Palliative care Outcome Scale. Palliat Med. 2016;30(6):599–610. doi:10.1177/0269216315608348 PubMed PMID: 26415736. 10.1177/0269216315608348 PMC4873725 26415736

[89] Schmidt H, Brandt D, Bischof A, Heidbrink S, Bischof G, Borgwardt S, et al. Think-aloud analysis of commonly used screening instruments for Internet use disorders: The CIUS, the IGDT-10, and the BSMAS. J Behav Addict. 2022;11(2):467–80. 10.1556/2006. 2022.00034 PubMed PMID: 35895610. PMC9295229 35895610

[90] Sheldon EM, Lillington G, Simpson K, Gibson K, Chambers L, D’Afflitto M, et al. Development of an inflammatory bowel disease (IBD) Patient-Reported Experience Measure (PREM): A patient-led consensus work and ‘think aloud’ study for a quality improvement programme. Health Expect. 2023;26(1):213–25. 10.1111/hex.13647 . PubMed PMID: 36335578. PMC9854292 36335578

[91] Solli R, Kvæl LAH, Olsen NR, Brovold T. Evaluation of content validity and feasibility of the World Falls Guidelines’ three key questions to identify falls among older adult users of home care services in Norway. BMC Health Serv Res. 2025;25(1). 10.1186/s12913-025-12606-y . PubMed PMID: 40148859. PMC11948927 40148859

[92] Sopfe J, Marsh R, Ziniel SI, Klosky JL, Chow EJ, Holliman BD, et al. Evaluation of the v2.0 brief profiles for sexual function and satisfaction PROMIS in adolescent and young adult childhood cancer survivors. J Adolesc Young Adult Oncol. 2021;10(4):418–24. 10.1089/jayao.2020.0166 . PubMed PMID: 33136468. 33136468

[93] Sussex AK, Rencz F, Gaydon M, Lloyd A, Gallop K. Exploring the content validity of the EQ-5D-5L and four bolt-ons (skin irritation, self-confidence, sleep, social relationships) in atopic dermatitis and chronic urticaria. Qual Life Res. 2025;34(4):991–1002. 10.1007/s11136-024-. 03875-x PubMed PMID: 39673587. PMC11982113 39673587

[94] Szlávicz E, Szabó Á, Kinyó Á, Szeiffert A, Bancsók T, Brodszky V, et al. Content validity of the EQ-5D-5L with skin irritation and self-confidence bolt-ons in patients with atopic dermatitis: a qualitative think-aloud study. Qual Life Res. 2024;33(1):101–11. 10.1007/s11136-023-03519-6 . PubMed PMID: 37787930. PMC10784357 37787930

[95] Tatlock S, Bentley S, Arbuckle R, Abetz-Webb L, Scott J. Content validation of a caregiver diary to monitor severity and recovery of pediatric patients with respiratory syncytial virus infection. J Patient Rep Outcomes. 2022;6(1). 10.1186/s41687-022-00442-1 . PubMed PMID: 35554751. PMC9098775 35554751

[96] Thompson C, Adams J, Vidgen HA. Progressing the development of a food literacy questionnaire using cognitive interviews. Public Health Nutr. 2022;25(7):1968–78. 10. 1017/S1368980021004560 PubMed PMID: 34743775. 10.1017/S1368980021004560 PMC9991617 34743775

[97] Thorvaldsdottir KB, Halldorsdottir S, Johnson RM, Sigurdardottir S, Saint Arnault D. Adaptation of the barriers to help-seeking for trauma (BHS-TR) scale: a cross-cultural cognitive interview study with female intimate partner violence survivors in Iceland. J Patient Rep Outcomes. 2021;5(1). 10.1186/s41687-021-00295-0 . PubMed PMID: 33638750. PMC7914310 33638750

[98] Van Leeuwen KM, Jansen APD, Muntinga ME, Bosmans JE, Westerman MJ, Van Tulder MW, et al. Exploration of the content validity and feasibility of the EQ-5D-3L, ICECAP-O and ASCOT in older adults. BMC Health Serv Res. 2015;15(1). 10.1186/s12913-. 015-0862-8 PubMed PMID: 25976227. PMC4435604 25976227

[99] Van Oort L, Schröder C, French DP. What do people think about when they answer the brief illness perception questionnaire? A think-aloud study. Br J Health Psychol. 2011;16(2):231–45. 10.1348/135910710X500819 . PubMed PMID: 21489052. 21489052

[100] Vreeman RC, Nyandiko WM, Ayaya SO, Walumbe EG, Inui TS. Cognitive interviewing for cross-cultural adaptation of pediatric antiretroviral therapy adherence measurement items. Int J Behav Med. 2014;21(1):186–96. 10.1007/s12529-012-9283-9 . PubMed PMID: 23188670. 23188670

[101] Wang X, Gao S, Wang T, Xue J, Yang Y, Han L, et al. Cognitive interviewing validation of the Chinese version of the neurogenic bladder symptom score. Heliyon. 2024;10(18). 10.1016/j.heliyon.2024.e37435. PMC11414480 39309897

[102] Watanabe S, Nekolaichuk C, Beaumont C, Mawani A. The Edmonton symptom assessment system-what do patients think? Support Care Cancer. 2009;17(6):675–83. 10.1007/s00520-008-0522-1 . PubMed PMID: 18953577. 18953577

[103] Zarnowiecki D, Byrne RA, Bodner GE, Bell LK, Golley RK. Improving the reporting of young children’s food intake: Insights from a cognitive interviewing study with mothers of 3–7-year old children. Nutrients. 2020;12(6). 10.3390/nu12061645 . PubMed PMID: 32498296. PMC7352554 32498296

[104] Patrick DL, Burke LB, Gwaltney CJ, Leidy NK, Martin ML, Molsen E, et al. Content validity - Establishing and reporting the evidence in newly developed patient-reported outcomes (PRO) instruments for medical product evaluation: ISPOR PRO good research practices task force report: Part 1 - Eliciting concepts for a new PRO instrument. Value Health. 2011;14(8):967–77. 10.1016/j.jval.2011.06.014 . PubMed PMID: 22152165. 22152165

[105] Nielsen MG, Lyng KD, Holden S, Johansen SK, Winters M, Rathleff MS. Item generation for a new patient-reported outcome measure: The non-traumatic anterior knee pain (AKP)-YOUTH scale. Musculoskelet Sci Pract. 2024;73:103151. 10.1016/j.msksp.2024.103151 . PubMed PMID: 2033518508. 39068686

[106] Lewis JR. Sample Sizes for Usability Studies: Additional Considerations. HUMAN FACTORS.1994;36(2):368–78. 10.1177/001872089403600. 8070799

[107] Caulton DA. Relaxing the homogeneity assumption in usability testing. Behav Inform Technol. 2001;20(1):1–7. 10.1080/01449290010020648.

[108] Villamin P, Lopez V, Thapa DK, Cleary M. A Worked Example of Qualitative Descriptive Design: A Step-by-Step Guide for Novice and Early Career Researchers. J Adv Nurs. 2025;81(8):5181–95. 10.1111/jan.16481 . PubMed PMID: 39382252. PMC12271654 39382252

[109] Van Den Haak MJ, De Jong MDT, Schellens PJ. Retrospective vs. concurrent think-aloud protocols: Testing the usability of an online library catalogue. Behav Inform Technol. 2003;22(5):339–51. 10.1080/0044929031000.

[110] Ericsson KASHA. Protocol Analysis: Verbal Reports as Data. Cambridge, MA: MIT Press; 1984.

[111] Ted Boren - M, Ramey J. Thinking Aloud: Reconciling Theory and Practice. IEEE TRANSACTIONS ON PROFESSIONAL COMMUNICATION. 2000;43(3):261–8. 10.1109/47.867942.

[112] Tarantini MG, Ramos S, Secrétan PH, Guichard L, Hassani L, Bellanger A, et al. Therapeutic Treatment Plan Optimization during the COVID-19 Pandemic: A Comprehensive Physicochemical Compatibility Study of Intensive Care Units Selected Drugs. Pharmaceutics. 2022;14(3). 10.3390/pharmaceutics14030550. PMC8952813 35335926

[113] Garvey CM, Jones R. Is There a Place for Theoretical Frameworks in Qualitative Research? Int J Qual Methods. 2021;20. 10.1177/1609406920987959.

[114] Poses RM, Isen AM. Qualitative Research in Medicine and Health Care Questions and Controversy. 1998;13(1):32–8. 10.1046/j.1525-1497.1998.00005.x. PMC1496891 9462492

[115] Dunivin ZO. Scalable Qualitative Coding with LLMs: Chain-of-Thought Reasoning Matches Human Performance in Some Hermeneutic Tasks. 2024. Available from: http://arxiv.org/abs/2401.15170.

