# Supplementary material for: Thinking-aloud methodology applied to health questionnaire development and validation: a scoping review
Source: BMC Med Res Methodol. 2026 Jun 20;26:217. doi: 10.1186/s12874-026-02927-9 (PMC13640185; doi:10.1186/s12874-026-02927-9)
Supplement: Supplementary file 1 [file 12874_2026_2927_MOESM1_ESM.docx]

**Supplementary file n.1, Search strategy**

**Search string for Pubmed**

*("Surveys and Questionnaires"[Mesh] OR "Psychometrics"[Mesh] OR "Validation Studies as Topic"[Mesh] OR "Interviews as Topic"[Mesh] OR valid*[Title/Abstract] OR verif*[Title/Abstract] OR questionnaire*[Title/Abstract] OR survey*[Title/Abstract] OR assess*[Title/Abstract] OR interview*[Title/Abstract]) AND ("think* aloud"[Title/Abstract]).*

**Search string for Scopus**

(TITLE-ABS(valid*) OR TITLE-ABS(verif*) OR INDEXTERMS("Validation Studies as Topic")) AND (INDEXTERMS("Surveys and Questionnaires") OR INDEXTERMS(Psychometrics) OR INDEXTERMS("Interviews as Topic") OR TITLE-ABS(questionnaire*) OR TITLE-ABS(survey*) OR TITLE-ABS(assess*) OR TITLE-ABS(interview*)) AND (TITLE-ABS("think* aloud")).

**Search string for Embase**

*(exp "validation study"/ or valid*.ti,ab. or verif*.ti,ab.)* ***and*** *(exp questionnaire/ or exp psychometry/ or exp interviews/ or questionnaire*.ti,ab. or survey*.ti,ab. or assess*.ti,ab. or interview*.ti,ab.)* ***and*** *("think* aloud".ti,ab.)*
