# Supplementary material for: Thinking-aloud methodology applied to health questionnaire development and validation: a scoping review
Source: BMC Med Res Methodol. 2026 Jun 20;26:217. doi: 10.1186/s12874-026-02927-9 (PMC13640185; doi:10.1186/s12874-026-02927-9)
Supplement: Supplementary file 2 [file 12874_2026_2927_MOESM2_ESM.docx]

| Author | **Year** | **Journal** | **Country** | **Research area** | **Instrument type tested (questionnaire name)** | **Stated purpose of the included studiesStated purpose of the included studies** | **Stated purpose of thinking-aloud methodology** | **Reference** |
| --- | --- | --- | --- | --- | --- | --- | --- | --- |
| Alberts | 2020 | BMJ Open | Germany | Medicine | TEX-Q | Developing and validating a questionnaire | To test comprehension and clarity | (1) |
| Al-Ebrahim | 2024 | Exploratory research in clinical and social pharmacy | New Zeland/Australia/Emirates | Pharmacology | MRB-QoL | Translating, culturally adapting and validating a questionnaire | Content validation of the questionnaire | (2) |
| Andersen | 2014 | International journal of nursing studies | Norway/Sweden | Medicine | COMFORT scale | Translating, culturally adapting and validating a questionnaire | To test comprehension and clarity | (3) |
| Andersson | 2022 | International Journal of Environmental Research and Public Health | Sweden | Medicine | PCSE scale | Translating, culturally adapting and validating a questionnaire | Content validation of the questionnaire | (4) |
| Augusto | 2014 | Revista Latino-Americana de Enfermagem | Portugal | Nursing | IFPF | Translating, culturally adapting and validating a questionnaire | Content validation of the questionnaire | (5) |
| Avallin | 2023 | Journal of advanced Nursing. | Australia | Nursing | PCPM | Developing and validating a questionnaire | For testing acceptability and feasibility | (6) |
| Bailey | 2015 | Journal of Clinical Pharmacy and Therapeutics | United Kingdom | Pharmacy | Antimicrobial Self-Assessment Toolkit for NHS Trusts (ASAT v15a) | Validating the content of a questionnaire | To test comprehension and clarity | (7) |
| Balstad | 2019 | Patient Preference and Adherence | Norway | Medicine | Patient-Generated Subjective Global Assessment Short Form (PG-SGA SF) | Validating the content of a questionnaire | To test comprehension and clarity | (8) |
| Barais | 2018 | BMJ Open | Nederland | Medicine | GFQ | Developing and validating a questionnaire | Content validation of the questionnaire | (9) |
| Bear | 2024 | Counselling Psychology Quarterly | United Kingdom | Psychology | IPQ Anxiety and IPQ Depression | Developing and validating a questionnaire | Content validation of the questionnaire | (10) |
| Benson | 2016 | Journal of Sport & Exercise Psychology | Canada | Psychology | STSTQ | Developing and validating a questionnaire | Content validation of the questionnaire | (11) |
| Bentley | 2021 | Journal of Patient- Reported Outcomes | US/Germany/France | Medicine | SAQ-AE (Scottish Staff Safety Attitudes and Adverse Events Questionnaire) | Validating the content of a questionnaire | Content validation of the questionnaire | (12) |
| Blackmore | 2024 | VALUE HEALTH | Australia | Medicine | EQ-5D-Y-5L (proxy-report version) | Further validating an already finalized questionnaire | For pilot and exploratory testing of the questionnaire | (13) |
| Blazeby | 2024 | Colorectal disease | United Kingdom | Medicine | PSH | Developing and validating a questionnaire | For face validation | (14) |
| Boome | 2022 | European Journal of Surgical Oncology |  | Medicine | ABCRC | Further validating an already finalized questionnaire | Other | (15) |
| Bos | 2019 | Journal of Pediatric Rehabilitation Medicine | The netherlands | Medicine | PEDI-CAT | Translating, culturally adapting and validating a questionnaire | Content validation of the questionnaire | (16) |
| Brod | 2023 | Quality of life research | USA | Medicine | F‑36v2 | Validating the content of a questionnaire | Other | (17) |
| Broderick | 2025 | Journal of patient-Reported Outcomes | USA | Medicine | Angioedema  Quality of Life Questionnaire (AE-QoL) | Validating the content of a questionnaire | Content validation of the questionnaire | (18) |
| Brown | 2009 | Journal of Anxiety Disorders | United Kingdom | Medicine/PSYCHOLOGICAL | ASI - Anxiety Sensitivity Index | Validating the construct | To test comprehension and clarity | (19) |
| Brunius Enlund | 2019 | PLOS ONE | Sweden | Veterinary science | Dental home care and dental health in Swedish dog | Developing and validating a questionnaire | For face validation | (20) |
| Brust | 2022 | Preventive Medicine Reports | Germany | Medicine | CardiacTM and CardiacLCI | Developing and validating a questionnaire | For pilot and exploratory testing of the questionnaire | (21) |
| Burrell | 2018 | British Journal of Health Psychology | UK/USA | Psychology | DCV | Validating the content of a questionnaire | Content validation of the questionnaire | (22) |
| Byrne | 2023 | Children | Australia | Psychology | MBQ-B; MBQ-C | Further validating an already finalized questionnaire | To test comprehension and clarity | (23) |
| Caiels | 2025 | Quality of life research | Uk | Social care | ASCOT -E R (Easy Read version of the Adult Social Care Outcomes Toolkit) | Validating the content of a questionnaire | To test comprehension and clarity | (24) |
| Campos | 2016 | Universitas Psychologica | Portugal | Psychology | MHLq | Developing and validating a questionnaire | Content validation of the questionnaire | (25) |
| Carnlof | 2020 | Nordic Journal of Nursing Research | Stockholm | Nursing | SCL | Translating, culturally adapting and validating a questionnaire | Content validation of the questionnaire | (26) |
| Cecchetto | 2021 | Jornal de Pediatria | Brazil | Nursing | LKQCHD | Translating, culturally adapting and validating a questionnaire | To test comprehension and clarity | (27) |
| Cella | 2013 | Gynecologic Oncology | USA | Medicine | FACIT AI | Further validating an already finalized questionnaire | Content validation of the questionnaire | (28) |
| Chua | 2021 | Annals of Palliative Medicine | Singapore | Medicine | CODE EM | Developing and validating a questionnaire | Content validation of the questionnaire | (29) |
| Closson | 2020 | BMC Public Health | Canada | Psychological / Psychiatric Care / Sexual and Reproductive Health | SRPS - Sexual Relationship Power  Scale | Validating the content of a questionnaire | To test comprehension and clarity | (30) |
| Coats | 2018 | Applied Nursing Research | USA | Nursing | Psychological-Social-Spiritual Healing instrument | Validating the content of a questionnaire | To test comprehension and clarity | (31) |
| Coleman | 2016 | BMC Medical Research Methodology | Uk | Nursing | PURPOSE-T (Pressure Ulcer Risk Primary Or Secondary Evaluation Tool) | Further validating an already finalized questionnaire | To test comprehension and clarity | (32) |
| Coombes | 2024 | Palliative Medicine | Uk |  | C-POS | Further validating an already finalized questionnaire | Other | (33) |
| Denovan | 2024 | Frontiers in Psychology | Turkiye | Psychology | PHBS | Further validating an already finalized questionnaire | To test comprehension and clarity | (34) |
| Despina | 2023 | MC Palliative Care | Grece | Medicine | IPOS tool | Translating, culturally adapting and validating a questionnaire | For pilot and exploratory testing of the questionnaire | (35) |
| Dias | 2018 | International Journal of Environmental research and Public Health | Portugal | Medicine | MHLq | Further validating an already finalized questionnaire | For pilot and exploratory testing of the questionnaire | (36) |
| Dornoville De la Cour | 2021 | Frontiers in Human Neurosciences | Denmark | Psychology | DMFS | Translating, culturally adapting and validating a questionnaire | To test comprehension and clarity | (37) |
| Drakenberg | 2023 | PLOS ONE | Sweden | Social care | Family Involvement in Care Questionnaire (FICQ) | Developing and validating a questionnaire | To test comprehension and clarity | (38) |
| Drolette - Labelle | 2024 | Public Health Nutrition | Canada | Nutrition | PBP-FFQ | Developing and validating a questionnaire | Content validation of the questionnaire | (39) |
| Duc | 2016 | Global Health Action | Sweden | Social care | COACH | Further validating an already finalized questionnaire | Content validation of the questionnaire | (40) |
| Eardley | 2012 | Forschende Komplementarmedizin | Uk | Medicine | CAM | Translating, culturally adapting and validating a questionnaire | Content validation of the questionnaire | (41) |
| Edwards | 2005 | Field methods | Sweden | Social sciences |  | Developing and validating a questionnaire | Content validation of the questionnaire | (42) |
| Egger-Rainer | 2019 | Research methodology | Austria | nursing | EMUCQ | Further validating an already finalized questionnaire | For testing acceptability and feasibility | (43) |
| Eland | 2019 | Physioteraphy theory and practice | Norway | Physiotheraphy | PABS | Validating the content of a questionnaire | Other | (44) |
| Eldh | 2014 | Health expectations | Sweden | Medicine | 4Ps | Developing and validating a questionnaire | Content validation of the questionnaire | (45) |
| Engel | 2023 | The patient centered outcome research | Australia | qualitative methodology, psychometrics, and health economics. | EQ-5D-5L, EQ-HWB, ASCOT, QOL-ACC | Testing the face validity | For face validation | (46) |
| Epstein | 2019 | Child care health development | Australia | Medicine | QI - Disability | Developing and validating a questionnaire | Content validation of the questionnaire | (47) |
| Ernstmann | 2017 | ZEFQ | Germany | Medicine | HL-COM | Developing and validating a questionnaire | For pilot and exploratory testing of the questionnaire | (48) |
| Evans | 2016 | Violence and Victims | Uk | Social sciences | CAS | Validating the content of a questionnaire | For face validation | (49) |
| Findley | 2023 | Journal of patient-Reported Outcomes | USA and Germany | Medicine | PROMIS | Validating the content of a questionnaire | Content validation of the questionnaire | (50) |
| Fishleder | 2022 | Frontiers in public health | USA | Social sciences | CCL self assessment | Developing and validating a questionnaire | For face validation | (51) |
| Gabes | 2022 | British Journal of Dermatology | Germany and The Netherlands | Medicine | RECAP | Validating the content of a questionnaire | Content validation of the questionnaire | (52) |
| Gabes | 2021 | Journal of patient-Reported Outcomes | Germany | Medicine | RECAP | Translating, culturally adapting and validating a questionnaire | Content validation of the questionnaire | (53) |
| Gadermann | 2011 | Social Indicators Research | Canada | Psychological / Educational | SWLS-C | Validating the construct | For testing acceptability and feasibility | (54) |
| Gao | 2019 | BMC Health Services Research | Uk | Psychology | PAM-13 | Developing and validating a questionnaire | Content validation of the questionnaire | (55) |
| Garfield | 2021 | BMC Health Services Research | Uk | Medicine | MODRum | Validating the content of a questionnaire | Content validation of the questionnaire | (56) |
| Gerlach | 2020 | BMC Cancer | Germany | Medicine | MyPOS | Translating, culturally adapting and validating a questionnaire | To test comprehension and clarity | (57) |
| Giles | 2013 | BMJ Qual Saf | Uk | Medicine | PMOS | Developing and validating a questionnaire | For face validation | (58) |
| Gohil | 2024 | Journal of vascular nursing | India | Nursing | TPB | Translating, culturally adapting and validating a questionnaire | For face validation | (59) |
| Gram | 2016 | Journal of Human Development and Capabilities | Uk | Social Sciences | Relative Autonomy Index | Other | Content validation of the questionnaire | (60) |
| Granat | 2022 | BMC Palliative Care | Sweden | Medicine | SEPC | Translating, culturally adapting and validating a questionnaire | For face validation | (61) |
| Hagen | 2008 | Journal of Pain and Symptom managment | Canada | Medicine | Alberta Breakthrough Pain Assessment Tool | Validating the construct | Content validation of the questionnaire | (62) |
| Haker | 2021 | Multiple Sclerosis and Related Disorders | Germany | Medicine | PBMS-R | Developing and validating a questionnaire | For pilot and exploratory testing of the questionnaire | (63) |
| Hancox | 2015 | Journal of dance medicine &Science | Uk | Medicine | PMCSQ | Validating the content of a questionnaire | Content validation of the questionnaire | (64) |
| Hanif | 2025 | Pakistani Armed Force Medical Journal | Pakistan | Medicine | HICAT | Further validating an already finalized questionnaire | Other | (65) |
| Hanning | 2009 | Revue canadienne de la pratique et de la recherche en diététique | Canada | Nutrition | FBQ | Further validating an already finalized questionnaire | Other | (66) |
| Hartmann-Boyce | 2016 | Obesity | Uk | Medicine | OxFAB | Developing and validating a questionnaire | For face validation | (67) |
| Hartmann-Boyce | 2022 | Obesity | Uk | Medicine | Shortened OxFAB | Developing and validating a questionnaire | For face validation | (68) |
| Hauge | 2015 | Qualitative health research | USA | Medicine | CHOICES | Other | Content validation of the questionnaire | (69) |
| Hebert | 2012 | Archives of Gerontology and Geriatrics | Canada | Medicine | SMAF | Developing and validating a questionnaire | For pilot and exploratory testing of the questionnaire | (70) |
| Hennel | 2020 | BMC Medical Education | Swizerland | Medicine | MSF-RG | Developing and validating a questionnaire | Content validation of the questionnaire | (71) |
| Hennrich | 2022 | BMC Health Services Research | Germany | Medicine | NCQ | Translating, culturally adapting and validating a questionnaire | Content validation of the questionnaire | (72) |
| Henry | 2022 | Supportive Care in Cancer | Australia | Psychology | CLEO | Developing and validating a questionnaire | Content validation of the questionnaire | (73) |
| Hernan | 2015 | BMJ Quality and Safety Online | Australia | Medicine | PC PMOS | Developing and validating a questionnaire | For face validation | (74) |
| Hildingsson | 2017 | Women and Birth | Sweden | Medicine | FOBS | Further validating an already finalized questionnaire | For testing acceptability and feasibility | (75) |
| Hoffman | 2019 | Measurement in Physical Education and Exercise Science |  | Medicine | AMQ | Developing and validating a questionnaire | Content validation of the questionnaire | (76) |
| Hornsey | 2021 | BMJ Open | Uk | Medicine | Acne Qol | Developing and validating a questionnaire | Other | (77) |
| Horwood | 2014 | Applied research quality of life | Uk | Medicine | ICECAP-O | Developing and validating a questionnaire | For face validation | (78) |
| Horwood | 2010 | BMC Muscoloskeletal Disorders | Uk | Medicine | Ab-IAP | Further validating an already finalized questionnaire | For testing acceptability and feasibility | (79) |
| Hudon | 2019 | Plos ONE | Canada | Medicine | PIH | Translating, culturally adapting and validating a questionnaire | For pilot and exploratory testing of the questionnaire | (80) |
| Humphrey | 2013 | Health and quality of life outcomes |  | Medicine | CBQ-HF | Testing the face validity | For face and content validation | (81) |
| Huusko | 2018 | Nursing research and Practice | Sweden | Midewifery | FaPPS | Further validating an already finalized questionnaire | For testing acceptability and feasibility | (82) |
| Igwesi‑Chidobe | 2024 | Journal of Occupational Rehabilitation | Nigeria | Medicine | ORFQ | Translating, culturally adapting and validating a questionnaire | For face and content validation | (83) |
| Igwesi‑Chidobe | 2021 | Journal of Patient - Reported Outcome | Nigeria | Medicine | CSQ | Translating, culturally adapting and validating a questionnaire | For face and content validation | (84) |
| Iqbal | 2024 | Frontiers in Medicine | Canada | Medicine | SJT | Validating the construct | To test comprehension and clarity | (85) |
| Jeannis | 2019 | Disability and Rehabilitation: Assistive technology | USA | Medicine | FPSEA | Developing and validating a questionnaire | Content validation of the questionnaire | (86) |
| Jefferson | 2017 | BRJ | UK | Medicine | PROFHER | Developing and validating a questionnaire | Other | (87) |
| Jeffrey | 2024 | The Journal of Sex Research | UK | Psychology | SES-SFV | Validating the content of a questionnaire | To test comprehension and clarity | (88) |
| Kabboord | 2019 | Clinical Interventions in Aging | the Netherlands | Medicine | w-FCI | Developing and validating a questionnaire | For testing acceptability and feasibility | (89) |
| Kaklamanou | 2013 | The British Psychological Society | UK | Psychology | Full compensatory health belief scale | Further validating an already finalized questionnaire | For face validation | (90) |
| Kaltenbrunner | 2017 | BMC Health Services Research | Sweden | Medicine | LiHcQ | Further validating an already finalized questionnaire | For face validation | (91) |
| Kaltebrunner | 2024 | BMC Health Services Research | Sweden | Medicine | Swe-IPAT | Translating, culturally adapting and validating a questionnaire | For pilot and exploratory testing of the questionnaire | (92) |
| Karimijashni | 2024 | Disability and Rehabilitation | Canada | Physioterapy | OA | Developing and validating a questionnaire | For face and content validation | (93) |
| Kassam | 2017 | BMC Palliative Care | USA | Nursing | BACPACS | Developing and validating a questionnaire | Content validation of the questionnaire | (94) |
| Khanna | 2024 | Health and Quality of life outcome | Australia | Medicine | EQ-5D-5L | Further validating an already finalized questionnaire | Content validation of the questionnaire | (95) |
| Kiejna | 2020 | Psychiatria Polska | Poland | Psychology | SACS | Translating, culturally adapting and validating a questionnaire | For pilot and exploratory testing of the questionnaire | (96) |
| Kovacevic Lepojevic | 2024 | Sage Journals | Serbia | Psychology | MHLq-SVa | Further validating an already finalized questionnaire | Other | (97) |
| Krieger | 2020 | BMC Pediatrics | Germany | Social Sciences | YC-PEM | Translating, culturally adapting and validating a questionnaire | For pilot and exploratory testing of the questionnaire | (98) |
| Krieger | 2023 | Frontiers in Pediatrics | Zurich | Social Sciences | YC-PEM | Translating, culturally adapting and validating a questionnaire | For pilot and exploratory testing of the questionnaire | (99) |
| Krishna-Naik | 2024 | Journal of Dentistry | UK | Dentistry | SRPQ | Further validating an already finalized questionnaire | Content validation of the questionnaire | (100) |
| Laberge | 2021 | Canadial jounral of Respiratory,Critical Care, and Sleep Medicine | Canada | Medicine | COPD disaster preparedness Questionnaire | Developing and validating a questionnaire | To test comprehension and clarity | (101) |
| Lay | 2024 | Health and Quality of life outcome | Australia | Social Sciences | QOL-ACC | Developing and validating a questionnaire | Content validation of the questionnaire | (102) |
| Loof | 2012 | Journal of advanced nursing | Sweden | Nursing | Body Awareness Questionnaire | Developing and validating a questionnaire | For testing acceptability and feasibility | (103) |
| Manns | 2018 | Muscolokeletal Science and Practice | UK | Physiotherapy | BIoH | Further validating an already finalized questionnaire | To test comprehension and clarity | (104) |
| Marconi | 2023 | Health and Quality of life outcomes | Italy | Medicine | PedQL | Translating, culturally adapting and validating a questionnaire | For pilot and exploratory testing of the questionnaire | (105) |
| Marmura | 2024 | The American Journal of sport medicine | Canada | Medicine | KNEES-ACL | Translating, culturally adapting and validating a questionnaire | Content validation of the questionnaire | (106) |
| Martin | 2021 | Bladder Cancer | USA | Medicine | BPI-SF | Further validating an already finalized questionnaire | Content validation of the questionnaire | (107) |
| Mayland | 2014 | BMJ | UK | Medicine | CODE | Further validating an already finalized questionnaire | For face and content validation | (108) |
| McBurnie | 2023 | Addiction Research& theory | UK | Psychology | PTGI | Further validating an already finalized questionnaire | Content validation of the questionnaire | (109) |
| McClam | 2023 | BMC Health Services Research | USA | Management | FQHC | Further validating an already finalized questionnaire | For face and content validation | (110) |
| McCorry | 2014 | Psychology&Health | UK | Psychology | IPQ-R | Further validating an already finalized questionnaire | For testing acceptability and feasibility | (111) |
| McLoughlin | 2023 | Value in Health | Ireland |  | 5 QoL measures | Further validating an already finalized questionnaire | Content validation of the questionnaire | (112) |
| Medina-Porqueres | 2022 | Archive of bone and Joint Surgery | Spain | Medicine | VISA-H | Translating, culturally adapting and validating a questionnaire | For pilot and exploratory testing of the questionnaire | (113) |
| Mertens | 2017 | Pain Practice | Luxemburg | Medicine | CEQ | Further validating an already finalized questionnaire | Content validation of the questionnaire | (114) |
| Mills | 2024 | BMJ Pediatrics Open | UK | Medicine | PGSQ | Further validating an already finalized questionnaire | Other | (115) |
| Mitchell | 2020 | BMJ Open | UK | Medicine | EQ-5D-5L, ICECAP-A, ICECAP-O | Further validating an already finalized questionnaire | For testing acceptability and feasibility | (116) |
| Mitrofanova | 2021 | Eating and Weight Disorders | UK | Nutrition | ORTO-15 | Further validating an already finalized questionnaire | To test comprehension and clarity | (117) |
| Mohr | 2022 | International Journal of Environmental Research and Public Health | Germany | Medicine | AHP | Developing and validating a questionnaire | For face and content validation | (118) |
| Monaca | 2025 | Health Expecations | Germany | Medicine | PaPSC | Further validating an already finalized questionnaire | Content validation of the questionnaire | (119) |
| Monahan | 2019 | J Gen Intern Med | USA | Medicine | Symtrack | Developing and validating a questionnaire | Other | (120) |
| Moodley | 2019 | Plos One | South Africa | Medicine | AWACAN | Developing and validating a questionnaire | For face and content validation | (121) |
| Murphy | 2018 | BMC Health Services Research | UK | Medicine | PCOQ | Testing the face validity | For face validation | (122) |
| Murtagh | 2007 | Palliative Medicine | UK | Medicine | MSAS-SF,GDS-10,POS | Further validating an already finalized questionnaire | Other | (123) |
| Muthu | 2020 | Community dentistry and oral epidemiology | India | Dentistry | ECC | Developing and validating a questionnaire | Content validation of the questionnaire | (124) |
| Nafees | 2024 | Journal of Pediatric Surgery | Canada | Medicine | GOSH PREM | Translating, culturally adapting and validating a questionnaire | Other | (125) |
| Namisango | 2022 | Sage Journals | Uganda | Medicine | NIH-HEALS | Testing the face validity | For face validation | (126) |
| Ngorsuraches | 2007 | Research in social &administrative pharmacy | Thailand | Pharmacy | TRUST-Ph | Developing and validating a questionnaire | Content validation of the questionnaire | (127) |
| Nielsen | 2024 | Muscolokeletal Science and Practice | Denmark | Medicine | AKP-YOUTH | Developing and validating a questionnaire | For face validation | (128) |
| Nikolaus | 2019 | BMC Public Health | USA | Nutrition | FSSM | Further validating an already finalized questionnaire | Content validation of the questionnaire | (129) |
| NilssonKajermo | 2023 | Worl views on Evidence-based Nursing | Sweden | Nursing | CAI | Translating, culturally adapting and validating a questionnaire | For testing acceptability and feasibility | (130) |
| Nymark | 2017 | Cardiovascular Nursing | Sweden | Nursing | PA-AMI | Developing and validating a questionnaire | For testing acceptability and feasibility | (131) |
| Olsson | 2018 | BMC Pediatrics | Sweden | Medicine | PIPP-R | Translating, culturally adapting and validating a questionnaire | For face and content validation | (132) |
| Oosrerveld-Vlug | 2014 | Health and Quality of life Outcomes | the Netherlands | Medicine | MIDAM-LTC | Developing and validating a questionnaire | Other | (133) |
| Ossenberg | 2016 | Nurse Education today | Australia | Nursing | ANSAT | Developing and validating a questionnaire | For testing acceptability and feasibility | (134) |
| Paige | 2019 | Journal of Health Communication | USA | Medicine | TMeHL | Developing and validating a questionnaire | For testing acceptability and feasibility | (135) |
| Palmer | 2017 | Physiotherapy | UK | Physiotherapy | BIoH | Developing and validating a questionnaire | Content validation of the questionnaire | (136) |
| Pandina | 2021 | Health Quality Life Outcomes | USA | Psychology | ABI | Further validating an already finalized questionnaire | Content validation of the questionnaire | (137) |
| Panepinto | 2012 | Qual Life Res | USA | Medicine | PedsQLTM | Developing and validating a questionnaire | Content validation of the questionnaire | (138) |
| Park | 2021 | Journal of medical internet reasearch | USA | Nursing | DHLI | Further validating an already finalized questionnaire | Content validation of the questionnaire | (139) |
| Paterson | 2018 | Journal of health psychology | New Zeland | Psychology | IES | Further validating an already finalized questionnaire | Content validation of the questionnaire | (140) |
| Peitz | 2021 | Appetite | Germany | Psychology | MEI | Developing and validating a questionnaire | For pilot and exploratory testing of the questionnaire | (141) |
| Peled | 2018 | Brest | USA | Medicine | Nipple reconstruction scale | Developing and validating a questionnaire | Content validation of the questionnaire | (142) |
| Penton | 2022 | Patient reported outcomes | UK | Psychology | 4 questionnaires | Further validating an already finalized questionnaire | Content validation of the questionnaire | (143) |
| Pirjo | 2014 | BMC Psychology | Sweden | Psychology | Ilness Invalidation Inventory | Translating, culturally adapting and validating a questionnaire | Other | (144) |
| Pitulaj | 2020 | Advances in Clinical and Experimental Medicine | Poland | Medicine | DAS | Translating, culturally adapting and validating a questionnaire | Content validation of the questionnaire | (145) |
| Plazar | 2024 | Quality of life research | Hungary | Medicine | EQ-5D-5L | Further validating an already finalized questionnaire | Content validation of the questionnaire | (146) |
| Pool | 2010 | Manual Therapy | the Netherlands | Medicine | PCCL | Further validating an already finalized questionnaire | Content validation of the questionnaire | (147) |
| Prebble | 2022 | Scand J Caring Sci | Norway | Medicine | SA-SH-D | Further validating an already finalized questionnaire | Content validation of the questionnaire | (148) |
| Prior | 2011 | BMC Medical Research Methodology | UK | Medicine | AGQ | Developing and validating a questionnaire | For testing acceptability and feasibility | (149) |
| Quandt | 2009 | The journal of alternative and complementary medicine | USA | Medicine | I-CAM-Q | Developing and validating a questionnaire | For pilot and exploratory testing of the questionnaire | (150) |
| Rand | 2017 | Health and quality of life outcomes | UK | Social Sciences | ASCOT | Developing and validating a questionnaire | Content validation of the questionnaire | (151) |
| Renberg | 2008 | Journal of clinical pharmacy and therapeutics | UK | Pharmacy | PTRQoL | Further validating an already finalized questionnaire | For face and content validation | (152) |
| Rencz | 2022 | Quality of life research | Hungary | Medicine | EQ-5D | Further validating an already finalized questionnaire | Content validation of the questionnaire | (153) |
| Riekert | 2025 | Journal of cystic fibrosis | USA | Medicine | DCC | Developing and validating a questionnaire | Other | (154) |
| Rodrigues | 2022 | ARP | Portugal | Medicine | EARP | Translating, culturally adapting and validating a questionnaire | Other | (155) |
| Rodrigues | 2016 | Personality and Individual Differences | Portugal | Social Sciences | Morningness - Eveningness Quesionnaire | Translating, culturally adapting and validating a questionnaire | To test comprehension and clarity | (156) |
| Rogers | 2020 | Journal of Patient-Reported Outcomes | UK | Dentistry | CARIES-QC | Developing and validating a questionnaire | To test comprehension and clarity | (157) |
| Rohr | 2021 | Injury | Germany | Medicine | HRQOL | Further validating an already finalized questionnaire | Content validation of the questionnaire | (158) |
| Rotstein | 2022 | BMC Pregnancy and Childbirth | Sweden | Medicine | Karolinska Symptoms After Perineal Tear Inventory | Developing and validating a questionnaire | For face validation | (159) |
| Sadiqi | 2017 | European Spine Journal | the Netherlands | Medicine | AOSpine PROST | Developing and validating a questionnaire | For testing acceptability and feasibility | (160) |
| Saldaris | 2021 | Journal of Child Neurology | Australia | Medicine | CCSA | Further validating an already finalized questionnaire | Content validation of the questionnaire | (161) |
| Samuelyan | 2024 | European Journal of Clinical Pharmacology | Turkiye | Pharmacy | MPRAQ-TR | Further validating an already finalized questionnaire | For face validation | (162) |
| Schildmann | 2015 | Palliative Medicine | Germany | Medicine | Integrated palliative care Outcome Scale | Translating, culturally adapting and validating a questionnaire | For testing acceptability and feasibility | (163) |
| Sekhon | 2022 | BMC Health Services Research | UK | Psychology | TFA | Developing and validating a questionnaire | For pilot and exploratory testing of the questionnaire | (164) |
| Sheldon | 2023 | Health Expectations | UK | Medicine | IBD PREM | Further validating an already finalized questionnaire | For face and content validation | (165) |
| Sigvardosdotter | 2017 | International Journal of Environmental Research and Public Health | Sweden | Medicine | RTHC | Developing and validating a questionnaire | Content validation of the questionnaire | (166) |
| Simmonds | 2015 | Journal of Health Psychology | UK | Nutrition | 8 Item Self Efficacy for Healthy Diet Scale | Developing and validating a questionnaire | For testing acceptability and feasibility | (167) |
| Skevington | 2014 | Psychology&Health | UK | Psychology | WHOQOL-BREF | Further validating an already finalized questionnaire | For testing acceptability and feasibility | (168) |
| Solli | 2025 | BMC Health Services Research | Norway | Medicine | 3KQ | Developing and validating a questionnaire | Content validation of the questionnaire | (169) |
| Sopfe | 2021 | Journal of adolescent and young adult oncology | USA | Medicine | SexFS Brief | Further validating an already finalized questionnaire | For testing acceptability and feasibility | (170) |
| Sripaew | 2021 | BMC Palliative Care | Thailand | Medicine | SPICT-LIS | Translating, culturally adapting and validating a questionnaire | For pilot and exploratory testing of the questionnaire | (171) |
| Sussex | 2024 | Quality of life research | UK | Psychology | EQ-5D-5L | Further validating an already finalized questionnaire | Content validation of the questionnaire | (172) |
| Svantesson | 2014 | BMC Medical Ethics | Sweden | Medicine | Euro-MCD | Developing and validating a questionnaire | Content validation of the questionnaire | (173) |
| Sweet | 2024 | Spinal Cord | Canada | Medicine | SCI | Developing and validating a questionnaire | For face validation | (174) |
| Szlavicz | 2023 | Quality of life research | Hungary | Psychology | EQ-5D-5L | Further validating an already finalized questionnaire | Content validation of the questionnaire | (175) |
| Takasaki | 2022 | Phys Ther Sci | Japan | Medicine | Katakori Disability Index | Developing and validating a questionnaire | For testing acceptability and feasibility | (176) |
| Tatlock | 2022 | Journal of Patient Reported Outcome | UK | Medicine | PREOSORS | Further validating an already finalized questionnaire | Content validation of the questionnaire | (177) |
| Tatlock | 2015 | Value in Health | UK | Medicine | I-TAQ | Developing and validating a questionnaire | Content validation of the questionnaire | (178) |
| Taylor | 2017 | Psychology&Health | UK | Psychology | IPQ-R | Further validating an already finalized questionnaire | For face validation | (179) |
| Ternstrom | 2016 | Women and birth | Sweden | Medicine | FOBS | Further validating an already finalized questionnaire | Content validation of the questionnaire | (180) |
| Teusen | 2024 | BMJ Open | Germany | Psychology | PHQ-9 | Developing and validating a questionnaire | Other | (181) |
| Thermaenius | 2019 | Intensive &Critical Care Nursing | Sweden | Nursing | SFS-ICQ | Developing and validating a questionnaire | To test comprehension and clarity | (182) |
| Thompson | 2021 | Public Health Nutrition | Australia | Nutrition |  | Developing and validating a questionnaire | Content validation of the questionnaire | (183) |
| Thorvaldsdottir | 2021 | Journal of Patient-Reported Outcomes | Iceland | Psychology | BHS-TR | Translating, culturally adapting and validating a questionnaire | For pilot and exploratory testing of the questionnaire | (184) |
| Tremblay | 2020 | Healthcare | Canada | Medicine | P-SAFE | Translating, culturally adapting and validating a questionnaire | Other | (185) |
| Turner- Bowker | 2009 | Patient | USA | Medicine | HR-QOL | Developing and validating a questionnaire | Content validation of the questionnaire | (186) |
| Turner- Bowker | 2012 | Patient | USA | Medicine | HIT | Further validating an already finalized questionnaire | For pilot and exploratory testing of the questionnaire | (187) |
| Tyler | 2023 | Health Expectations | UK | Medicine | RMOS | Developing and validating a questionnaire | For pilot and exploratory testing of the questionnaire | (188) |
| van Leeuwen | 2015 | BMC Health Services Research | the Netherlands | Medicine | EQ-5D-3L,ICECAP-O and ASCOT | Developing and validating a questionnaire | Content validation of the questionnaire | (189) |
| van Leeuwen | 2015 | Health and quality of life outcomes | the Netherlands | Medicine | ASCOT SCT-4 | Translating, culturally adapting and validating a questionnaire | Content validation of the questionnaire | (190) |
| van Leeuwen | 2020 | Quality of life research | the Netherlands | Medicine | Ability to participate in social roles and activities | Further validating an already finalized questionnaire | Content validation of the questionnaire | (191) |
| van Melle | 2018 | Journal of Clinical Epidemiology | the Netherlands | Medicine | TRIQ | Developing and validating a questionnaire | For pilot and exploratory testing of the questionnaire | (192) |
| van Oort | 2011 | British journal of health Psychology | the Netherlands | Psychology | Brief IPQ | Further validating an already finalized questionnaire | Content validation of the questionnaire | (193) |
| van Stiphout | 2022 | Frontiers in Neurology | the Netherlands | Medicine | BVQ | Developing and validating a questionnaire | For face and content validation | (194) |
| Varni | 2012 | JPGN | USA | Medicine | PedsQL | Developing and validating a questionnaire | For testing acceptability and feasibility | (195) |
| Vaucher | 2012 | BMJ Open |  | Social sciences | DiPCare-Q | Developing and validating a questionnaire | For face validation | (196) |
| Veldhuijzen | 2020 | European Journal of Gastroenterology &Hepatology | the Netherlands | Medicine | GESQ | Translating, culturally adapting and validating a questionnaire | Other | (197) |
| Visser | 2024 | Clinical Nutrition | the Netherlands | Nutrition | MAS | Developing and validating a questionnaire | To test comprehension and clarity | (198) |
| vonScheven | 2024 | MDPI | USA | Medicine |  | Developing and validating a questionnaire | For face and content validation | (199) |
| Vreeeman | 2014 | Int. J. Behav. Med | USA | Medicine | CAMP | Translating, culturally adapting and validating a questionnaire | To test comprehension and clarity | (200) |
| Wang | 2024 | Heliyon | China | Medicine | NBSS | Translating, culturally adapting and validating a questionnaire | Content validation of the questionnaire | (201) |
| Watanabe | 2012 | Support Care Cancer | Canada | Medicine | ESAS | Further validating an already finalized questionnaire | Other | (202) |
| Watanabe | 2008 | Supportive Care in Cancer | Canada | Medicine | ESAS | Further validating an already finalized questionnaire | Content validation of the questionnaire | (203) |
| Wazqar | 2023 | Palliative and Supportive Care | Saudi Arabia | Nursing | PCSS | Translating, culturally adapting and validating a questionnaire | For pilot and exploratory testing of the questionnaire | (204) |
| Wenzel | 2024 | Multiple Sclerosis and Related Disorders | Germany | Nursing | RKQ | Developing and validating a questionnaire | For pilot and exploratory testing of the questionnaire | (205) |
| Wikilander | 2013 | Health and Quality of life outcome | Sweden | Medicine | HSS-40 | Further validating an already finalized questionnaire | Content validation of the questionnaire | (206) |
| Wu | 2020 | Quality of Life Research | China | Pharmacy | SF-6Dv2 | Translating, culturally adapting and validating a questionnaire | Content validation of the questionnaire | (207) |
| Zarnowiecki | 2020 | Nutrients | Australia | Nutrition | SFQ | Further validating an already finalized questionnaire | To test comprehension and clarity | (208) |
| Zhang | 2023 | Appetite | UK | Psychology | C-MEQ | Further validating an already finalized questionnaire | Other | (209) |
| Zuger | 2023 | BMJ Open | Germany | Medicine | ACPDS | Further validating an already finalized questionnaire | Other | (210) |

1.	Alberts J, Lowe B, Glahn MA, Petrie K, Laferton J, Nestoriuc Y, et al. Development of the generic, multidimensional Treatment Expectation Questionnaire (TEX-Q) through systematic literature review, expert surveys and qualitative interviews. BMJ Open [Internet]. 2020;10(8):e036169. Available from: http://bmjopen.bmj.com/content/early/by/section

2.	Al-Ebrahim SQ, Harrison J, Chen TF, Alzubaidi H, Mohammed MA. The Arabic medication-related burden quality of life (MRB-QoL) tool: Cross-cultural adaptation and content validation. Exploratory Research in Clinical and Social Pharmacy [Internet]. 2024;16:100523. Available from: https://www.sciencedirect.com/science/journal/26672766

3.	Andersen RD, Jylli L, Ambuel B. Cultural adaptation of patient and observational outcome measures: a methodological example using the COMFORT behavioral rating scale. Int J Nurs Stud [Internet]. 2014;51(6):934–42. Available from: https://ovidsp.ovid.com/ovidweb.cgi?T=JS&CSC=Y&NEWS=N&PAGE=fulltext&D=emed15&DO=10.1016%2fj.ijnurstu.2013.10.006

4.	Andersson S, Granat L, Brännström M, Sandgren A. Translation, Cultural Adaptation, and Content Validation of the Palliative Care Self-Efficacy Scale for Use in the Swedish Context. Int J Environ Res Public Health [Internet]. 2022;19(3). Available from: https://www.scopus.com/inward/record.uri?eid=2-s2.0-85122935875&doi=10.3390%2fijerph19031143&partnerID=40&md5=ff0e23a2e69e747f1afab27c1aa33b0e

5.	Augusto CC, Araujo BR, Rodrigues VM, Md.oC. de F. Adaptation and validation of the Inventory of Family Protective Factors for the Portuguese culture. Rev Lat Am Enfermagem [Internet]. 2014;22(6):1001–8. Available from: https://ovidsp.ovid.com/ovidweb.cgi?T=JS&CSC=Y&NEWS=N&PAGE=fulltext&D=emed15&DO=10.1590%2f0104-1169.3315.2509

6.	Avallin T, Jangland E, Kitson A, Muntlin A. Measuring person-centred pain management: Development of a questionnaire using the fundamentals of care framework. J Adv Nurs [Internet]. 2023;79(10):3923–34. Available from: https://ovidsp.ovid.com/ovidweb.cgi?T=JS&CSC=Y&NEWS=N&PAGE=fulltext&D=emed25&DO=10.1111%2fjan.15697

7.	Bailey C, Tully M, Cooke J. An investigation into the content validity of the Antimicrobial Self-Assessment Toolkit for NHS Trusts (ASAT v15a) using cognitive interviews with antimicrobial pharmacists. J Clin Pharm Ther [Internet]. 2015;40(2):208–12. Available from: http://onlinelibrary.wiley.com/journal/10.1111/(ISSN)1365-2710

8.	Balstad TR, Bye A, Jenssen CRS, Solheim TS, Thoresen L, Sand K. Patient interpretation of the patient-generated subjective global assessment (PG-SGA) short form. Patient Prefer Adherence. 2019;13:1391–400.

9.	Barais M, Van De Wiel MWJ, Groell N, Dany A, Montier T, Van Royen P, et al. Gut Feelings Questionnaire in daily practice: A feasibility study using a mixed-methods approach in three European countries. BMJ Open. 2018 Nov 1;8(11).

10.	Bear HA, Moon Z, Wasil A, Ahuvia I, Edbrooke-Childs J, Wolpert M. Development and validation of the illness perceptions questionnaire for youth anxiety and depression (IPQ-Anxiety and IPQ-Depression). Couns Psychol Q [Internet]. 2024;37(3):441–69. Available from: https://www.scopus.com/inward/record.uri?eid=2-s2.0-85164914620&doi=10.1080%2f09515070.2023.2232320&partnerID=40&md5=8418befac9c75356baae303be67ed30c

11.	Benson AJ, Eys M. Understanding the Consequences of Newcomer Integration Processes: The Sport Team Socialization Tactics Questionnaire. J Sport Exerc Psychol [Internet]. 2017;39(1):13–28. Available from: https://ovidsp.ovid.com/ovidweb.cgi?T=JS&CSC=Y&NEWS=N&PAGE=fulltext&D=emed18&DO=10.1123%2fjsep.2016-0182

12.	Tatlock S, Bentley S, Arbuckle R, Abetz-Webb L, Scott J. Content validation of a caregiver diary to monitor severity and recovery of pediatric patients with respiratory syncytial virus infection. J Patient Rep Outcomes [Internet]. 2022;6(1):48. Available from: https://ovidsp.ovid.com/ovidweb.cgi?T=JS&CSC=Y&NEWS=N&PAGE=fulltext&D=emed27&DO=10.1186%2fs41687-022-00442-1

13.	Blackmore AM, Mulhern B, Norman R, Reddihough D, Choong CS, Jacoby P, et al. How Well Does the EQ-5D-Y-5L Describe Children With Intellectual Disability?: “There’s a Lot More to My Child Than That She Can’t Wash or Dress Herself.” Value in Health [Internet]. 2024;27(2):190–8. Available from: https://www.sciencedirect.com/science/journal/10983015

14.	Blazeby JM, Murkin C, Rooshenas L, Elliott D, Avery K, Chalmers K, et al. Development and pilot testing of a patient-reported outcome measure to assess symptoms of parastomal hernia. Colorectal Disease [Internet]. 2024;26(2):364–70. Available from: http://onlinelibrary.wiley.com/journal/10.1111/(ISSN)1463-1318

15.	Boome IT, Somers AMJ, Graupner C, Kimman ML, M GSAH, Breukink SO. Development and content validation of the Assessment of Burden of ColoRectal Cancer (ABCRC)-tool. European Journal of Surgical Oncology [Internet]. 2022;48(8):1807–14. Available from: http://www.elsevier.com/inca/publications/store/6/2/3/0/3/3/index.htt

16.	Bos N, Engel MF, J VRN, Verheijden JMA, Coster W, Moed R, et al. Translation and cross-cultural adaptation of the PEDI-CAT: Dutch version. J Pediatr Rehabil Med [Internet]. 2019;12(1):57–64. Available from: http://iospress.metapress.com/content/120801/?p=e4b28739203a487a98e3ffa8f6e525e6&pi=54

17.	Brod M, Waldman LT, Shu AD, Smith A. Content validation of the SF-36v2 Health Survey Acute for use in hypoparathyroidism. Quality of Life Research [Internet]. 2023;32(6):1795–806. Available from: https://www.springer.com/journal/11136

18.	Broderick L, Foster A, Waldman LT, Jacobs KD, Bordone L, Yarlas A. Content validation of the Angioedema Quality of Life Questionnaire (AE-QoL) in a population of adult and adolescent patients with hereditary angioedema (HAE). J Patient Rep Outcomes. 2025 Dec 1;9(1).

19.	Brown GP, Hawkes NC, Tata P. Construct validity and vulnerability to anxiety: A cognitive interviewing study of the revised Anxiety Sensitivity Index. J Anxiety Disord [Internet]. 2009;23(7):942–9. Available from: https://ovidsp.ovid.com/ovidweb.cgi?T=JS&CSC=Y&NEWS=N&PAGE=fulltext&D=emed11&DO=10.1016%2fj.janxdis.2009.06.001

20.	Enlund KB, Brunius C, Hanson J, Hagman R, Höglund OV, Gustås P, et al. Development and validation of two questionnaires: Dental home care and dental health in Swedish dogs. PLoS One. 2019 Jan 1;14(1).

21.	Brust M, Gebhardt WA, van der Voorde NAE, Numans ME, Kiefte-de Jong JC. The development and validation of scales to measure the presence of a teachable moment following a cardiovascular disease event. Prev Med Rep. 2022 Aug 1;28.

22.	Burrell AMG, Allan JL, Williams DM, Johnston M. What do self-efficacy items measure? Examining the discriminant content validity of self-efficacy items. Br J Health Psychol [Internet]. 2018;23(3):597–611. Available from: https://ovidsp.ovid.com/ovidweb.cgi?T=JS&CSC=Y&NEWS=N&PAGE=fulltext&D=emed19&DO=10.1111%2fbjhp.12306

23.	Byrne R, Terranova CO, Chai LK, Brookes DSK, Trost SG. Cognitive Testing of Items Measuring Movement Behaviours in Young Children Aged Zero to Five Years: Development of the Movement Behaviour Questionnaires for -Baby (MBQ-B) and -Child (MBQ-C). Children. 2023 Sep 1;10(9).

24.	Caiels J, Rand S, Mikelyte R, Webster L, Field E, Towers AM. Enhancing quality of life measurement: adapting the ASCOT easy read for older adults accessing social care. Quality of Life Research. 2025 Jan 1;34(1):189–200.

25.	Campos L, Dias P, Palha F, Duarte A, Veiga E. Development and psychometric properties of a new questionnaire for assessing mental health literacy in young people. Universitas Psychologica [Internet]. 2016;15(2):61–72. Available from: https://www.scopus.com/inward/record.uri?eid=2-s2.0-85009461605&doi=10.11144%2fJaveriana.upsy15-2.dppq&partnerID=40&md5=8e5260e3d23112d40c7ff680ac66f210

26.	Carnlöf C, Malinowsky C, Insulander P, Gadler F, Jensen-Urstad M, Iwarzon M. The Symptom Checklist: Frequency and Severity Scale – translation and psychometric properties of the Swedish version. Nord J Nurs Res [Internet]. 2020;40(2):97–104. Available from: https://www.scopus.com/inward/record.uri?eid=2-s2.0-85107442325&doi=10.1177%2f2057158519898383&partnerID=40&md5=2b9c7a458888fb67d8e3efe372e48e0b

27.	Cecchetto FH, Bonato GD, Barreto TSM, Riegel F, Pellanda LC. Translation, cross-cultural adaptation, and validation of the Leuven Knowledge Questionnaire for congenital heart disease instrument into Brazilian Portuguese. J Pediatr (Rio J). 2021 Jul 1;97(4):402–8.

28.	Cella D, Neubauer N, Thomas J, Kutner J, Seiden M V. The FACIT-AI, a new tool for assessing symptoms associated with malignant ascites. Gynecol Oncol [Internet]. 2013;128(2):187–90. Available from: https://www.scopus.com/inward/record.uri?eid=2-s2.0-84872838620&doi=10.1016%2fj.ygyno.2012.11.013&partnerID=40&md5=061b8230dee1bbfc918bd32169e255f0

29.	Chua MT, W SK, Zheng CQ, Tiah L, Kumar R, Wong YKY, et al. Validation of “Care of the Dying Evaluation” in Emergency Medicine (CODE-EM): pilot phase of end-of-life management protocol offered within emergency room (EMPOWER) study. Ann Palliat Med [Internet]. 2021;10(6):6145–55. Available from: https://ovidsp.ovid.com/ovidweb.cgi?T=JS&CSC=Y&NEWS=N&PAGE=fulltext&D=emed23&DO=10.21037%2fapm-21-380

30.	Closson K, Zharima C, Kuchena M, Dietrich JJ, Gadermann A, Ogilvie G, et al. “I feel like it is asking if he is a stalker ... but I also feel like it is asking if he cares”: exploring young South African women and men’s perceptions of the Sexual Relationship Power Scale. BMC Public Health [Internet]. 2022;22(1):1368. Available from: https://ovidsp.ovid.com/ovidweb.cgi?T=JS&CSC=Y&NEWS=N&PAGE=fulltext&D=emed24&DO=10.1186%2fs12889-022-13686-9

31.	Coats H, Rosenfeld AG, Crist JD, Sternberg E, Berger A. Using cognitive interviews to improve a Psychological-Social-Spiritual Healing instrument: Voices of aging African Americans with serious illness. Applied Nursing Research [Internet]. 2018;39:109–14. Available from: https://www.scopus.com/inward/record.uri?eid=2-s2.0-85033361237&doi=10.1016%2fj.apnr.2017.11.019&partnerID=40&md5=84c94644609f65e4207a171de8f3143d

32.	Coleman S, Nixon J, Keen J, Muir D, Wilson L, McGinnis E, et al. Using cognitive pre-testing methods in the development of a new evidenced-based pressure ulcer risk assessment instrument. BMC Med Res Methodol [Internet]. 2016;16(1):158. Available from: https://ovidsp.ovid.com/ovidweb.cgi?T=JS&CSC=Y&NEWS=N&PAGE=fulltext&D=emed17&AN=618906022

33.	Coombes L, Braybrook D, Hardardottir D, Scott HM, Bristowe K, Ellis-Smith C, et al. Cognitive testing of the Children’s Palliative Outcome Scale (C-POS) with children, young people and their parents/carers. Palliat Med [Internet]. 2024;38(6):644–59. Available from: https://journals.sagepub.com/home/PMJ

34.	Denovan A, Dagnall N, Drinkwater KG. The paranormal health beliefs scale: an evaluation using cognitive interviewing. Front Psychol [Internet]. 2024;15. Available from: https://www.scopus.com/inward/record.uri?eid=2-s2.0-85185290532&doi=10.3389%2ffpsyg.2024.1306372&partnerID=40&md5=f94ed60b9f0ab6f2fbaa8df803fe0279

35.	Despina A, Stylianos K, Irene P, Elisabeth P, Aliki T. Translation and cultural adaptation of the Greek integrated palliative care outcome scale (IPOS): challenges in a six-phase process. BMC Palliat Care [Internet]. 2023;22(1):168. Available from: https://bmcpalliatcare.biomedcentral.com/

36.	Dias P, Campos L, Almeida H, Palha F. Mental health literacy in young adults: Adaptation and psychometric properties of the mental health literacy questionnaire. Int J Environ Res Public Health [Internet]. 2018;15(7):1318. Available from: http://www.mdpi.com/1660-4601/15/7/1318/pdf

37.	Dornonville de la Cour FL, Norup A, Schow T, Andersen TE. Evaluation of Response Processes to the Danish Version of the Dutch Multifactor Fatigue Scale in Stroke Using the Three-Step Test-Interview. Front Hum Neurosci. 2021 May 6;15.

38.	Drakenberg A, Sluys KP, Ericsson E, Sundqvist AS. The Family Involvement in Care Questionnaire —An instrument measuring family involvement in inpatient care. PLoS One. 2023 Aug 1;18(8 August).

39.	Drolet-Labelle V, Bedard A, Lemieux S, Drapeau V, Vanderlee L, Laurin D, et al. Development and cognitive testing of a food-frequency questionnaire to assess intake of plant-based protein foods among older adults. Public Health Nutr [Internet]. 2024;28(1):e16. Available from: https://ovidsp.ovid.com/ovidweb.cgi?T=JS&CSC=Y&NEWS=N&PAGE=fulltext&D=emexb&DO=10.1017%2fS1368980024002052

40.	Duc DM, Bergstrom A, Eriksson L, Selling K, B TTH, Wallin L. Response process and test-retest reliability of the Context Assessment for Community Health tool in Vietnam. Glob Health Action [Internet]. 2016;9:31572. Available from: https://ovidsp.ovid.com/ovidweb.cgi?T=JS&CSC=Y&NEWS=N&PAGE=fulltext&D=emed17&DO=10.3402%2fgha.v9.31572

41.	Eardley S, Bishop FL, Cardini F, Santos-Rey K, Jong MC, Ursoniu S, et al. A pilot feasibility study of a questionnaire to determine European union-wide CAM use. Forsch Komplementarmed [Internet]. 2012;19(6):302–10. Available from: https://www.scopus.com/inward/record.uri?eid=2-s2.0-84873373130&doi=10.1159%2f000345839&partnerID=40&md5=29e05cc12d7a673eb99369251894e2e4

42.	Edwards M, Thomsen SC, Toroitich-Ruto C. Thinking Aloud to Create Better Condom Use Questions. Field methods [Internet]. 2005;17(2):183–99. Available from: https://www.scopus.com/inward/record.uri?eid=2-s2.0-84996253024&doi=10.1177%2f1525822X04271029&partnerID=40&md5=cc8b026058c3ede9258502960f667bb7

43.	Egger-Rainer A. Enhancing validity through cognitive interviewing. A methodological example using the Epilepsy Monitoring Unit Comfort Questionnaire. J Adv Nurs [Internet]. 2019;75(1):224–33. Available from: https://ovidsp.ovid.com/ovidweb.cgi?T=JS&CSC=Y&NEWS=N&PAGE=fulltext&D=emed20&DO=10.1111%2fjan.13867

44.	Eland ND, Strand LI, Ostelo RW, Kvale A, Magnussen LH. How do physiotherapists understand and interpret the “Pain Attitudes and Beliefs Scale”? A cognitive interview study. Physiother Theory Pract [Internet]. 2022;38(4):513–27. Available from: https://ovidsp.ovid.com/ovidweb.cgi?T=JS&CSC=Y&NEWS=N&PAGE=fulltext&D=emed23&DO=10.1080%2f09593985.2020.1774949

45.	Eldh AC, Luhr K, Ehnfors M. The development and initial validation of a clinical tool for patients’ preferences on patient participation--The 4Ps. Health Expect [Internet]. 2015;18(6):2522–35. Available from: https://ovidsp.ovid.com/ovidweb.cgi?T=JS&CSC=Y&NEWS=N&PAGE=fulltext&D=emed16&DO=10.1111%2fhex.12221

46.	Engel L, Kosowicz L, Bogatyreva E, Batchelor F, Devlin N, Dow B, et al. Face Validity of Four Preference-Weighted Quality-of-Life Measures in Residential Aged Care: A Think-Aloud Study. Patient [Internet]. 2023;16(6):655–66. Available from: https://www.springer.com/journal/40271

47.	Epstein A, Williams K, Reddihough D, Murphy N, Leonard H, Whitehouse A, et al. Content validation of the Quality of Life Inventory-Disability. Child Care Health Dev [Internet]. 2019;45(5):654–9. Available from: https://ovidsp.ovid.com/ovidweb.cgi?T=JS&CSC=Y&NEWS=N&PAGE=fulltext&D=emed20&DO=10.1111%2fcch.12691

48.	Ernstmann N, Halbach S, Kowalski C, Pfaff H, Ansmann L. Measuring attributes of health literate health care organizations from the patients’ perspective: Development and validation of a questionnaire to assess health literacy-sensitive communication (HL-COM). Z Evid Fortbild Qual Gesundhwes [Internet]. 2017;121:58–63. Available from: https://ovidsp.ovid.com/ovidweb.cgi?T=JS&CSC=Y&NEWS=N&PAGE=fulltext&D=emed18&DO=10.1016%2fj.zefq.2016.12.008

49.	Evans M, Gregory A, Feder G, Howarth E, Hegarty K. “Even ‘daily’ is not enough”: How well do we measure domestic violence and abuse?-A think-aloud study of a commonly used self-report scale. Violence Vict [Internet]. 2016;31(1):3–26. Available from: https://www.scopus.com/inward/record.uri?eid=2-s2.0-84961644287&doi=10.1891%2f0886-6708.VV-D-15-00024&partnerID=40&md5=0f9ebad32c6693e13722322853a84092

50.	Findley A, Middlehurst JM, Howse CA, Clifford MJ, Neill W, Tatlock S, et al. Qualitative concept elicitation and cognitive debriefing interviews of symptoms, impacts and selected customized PROMIS® Short Forms: a study in patients with axial spondyloarthritis. J Patient Rep Outcomes. 2023 Dec 1;7(1).

51.	Fishleder S, Harris JR, Petrescu-Prahova M, Kohn M, Helfrich CD. Development and Feasibility Testing of the Clinical-Community Linkage Self-Assessment Survey for Community Organizations. Front Public Health [Internet]. 2022;10:797468. Available from: https://ovidsp.ovid.com/ovidweb.cgi?T=JS&CSC=Y&NEWS=N&PAGE=fulltext&D=emed23&DO=10.3389%2ffpubh.2022.797468

52.	Gabes M, Ragamin A, Baker A, Kann G, Donhauser T, Gabes D, et al. Content validity of the Recap of atopic eczema (RECAP) instrument in Dutch, English and German to measure eczema control in young people with atopic eczema: a cognitive interview study. British Journal of Dermatology [Internet]. 2022;187(6):919–26. Available from: http://onlinelibrary.wiley.com/journal/10.1111/(ISSN)1365-2133

53.	Gabes M, Tischer C, Herrmann A, Howells L, Apfelbacher C. The German RECAP questionnaire: linguistic validation and cognitive debriefing in German adults with self-reported atopic eczema and parents of affected children. J Patient Rep Outcomes [Internet]. 2021;5(1). Available from: https://www.scopus.com/inward/record.uri?eid=2-s2.0-85099747116&doi=10.1186%2fs41687-021-00285-2&partnerID=40&md5=cfa7e6106bc7e71adc9ac81cdb15412a

54.	Gadermann AM, Guhn M, Zumbo BD. Investigating the Substantive Aspect of Construct Validity for the Satisfaction with Life Scale Adapted for Children: A Focus on Cognitive Processes. Soc Indic Res [Internet]. 2011;100(1):37–60. Available from: https://www.scopus.com/inward/record.uri?eid=2-s2.0-78649877216&doi=10.1007%2fs11205-010-9603-x&partnerID=40&md5=37bea719f21ae9e0f4e4370353289714

55.	Gao J, Arden M, Hoo ZH, Wildman M. Understanding patient activation and adherence to nebuliser treatment in adults with cystic fibrosis: responses to the UK version of PAM-13 and a think aloud study. BMC Health Serv Res [Internet]. 2019;19(1):420. Available from: https://ovidsp.ovid.com/ovidweb.cgi?T=JS&CSC=Y&NEWS=N&PAGE=fulltext&D=emed20&DO=10.1186%2fs12913-019-4260-5

56.	Garfield K, Husbands S, Thorn JC, Noble S, Hollingworth W. Development of a brief, generic, modular resource-use measure (ModRUM): cognitive interviews with patients. BMC Health Serv Res [Internet]. 2021;21(1):371. Available from: https://ovidsp.ovid.com/ovidweb.cgi?T=JS&CSC=Y&NEWS=N&PAGE=fulltext&D=emed22&DO=10.1186%2fs12913-021-06364-w

57.	Gerlach C, Taylor K, Ferner M, Munder M, Weber M, Ramsenthaler C. Challenges in the cultural adaptation of the German Myeloma Patient Outcome Scale (MyPOS): An outcome measure to support routine symptom assessment in myeloma care. BMC Cancer. 2020 Mar 23;20(1).

58.	Giles SJ, Lawton RJ, Din I, McEachan RRC. Developing a patient measure of safety (PMOS). BMJ Qual Saf [Internet]. 2013;22(7):554–62. Available from: http://qualitysafety.bmj.com/content/22/7/554.full.pdf

59.	Gohil MN, Muruganantham B, Raval M, Bearne LM. Translation, cross-cultural adaptation, and validation of a Gujarati version of a theory of planned behavior questionnaire that assesses walking treatment beliefs in people with intermittent claudication. J Vasc Nurs [Internet]. 2024;42(3):182–90. Available from: https://ovidsp.ovid.com/ovidweb.cgi?T=JS&CSC=Y&NEWS=N&PAGE=fulltext&D=emed27&DO=10.1016%2fj.jvn.2024.05.004

60.	Gram L, Morrison J, Sharma N, Shrestha B, Manandhar D, Costello A, et al. Validating an Agency-based Tool for Measuring Women’s Empowerment in a Complex Public Health Trial in Rural Nepal. J Human Dev Capabil [Internet]. 2017;18(1):107–35. Available from: https://www.scopus.com/inward/record.uri?eid=2-s2.0-84994558785&doi=10.1080%2f19452829.2016.1251403&partnerID=40&md5=808ae515c238e38f60843835fe6f65aa

61.	Granat L, Andersson S, Hadziabdic E, Brannstrom M, Sandgren A. Translation, adaptation, and validation of the Self-efficacy in Palliative Care scale (SEPC) for use in Swedish healthcare settings. BMC Palliat Care [Internet]. 2022;21(1):48. Available from: http://www.biomedcentral.com/bmcpalliatcare/

62.	Hagen NA, Stiles C, Nekolaichuk C, Biondo P, Carlson LE, Fisher K, et al. The Alberta Breakthrough Pain Assessment Tool for Cancer Patients: A Validation Study Using a Delphi Process and Patient Think-Aloud Interviews. J Pain Symptom Manage [Internet]. 2008;35(2):136–52. Available from: https://ovidsp.ovid.com/ovidweb.cgi?T=JS&CSC=Y&NEWS=N&PAGE=fulltext&D=emed10&DO=10.1016%2fj.jpainsymman.2007.03.016

63.	Haker M, Heesen C, Wenzel L, Kopke S, Rahn AC, Kasper J. Decision-making about corticosteroids in relapses of multiple sclerosis - development of a questionnaire based on the theory of planned behaviour. Mult Scler Relat Disord [Internet]. 2021;55:103182. Available from: http://www.elsevier.com/wps/find/journaldescription.cws_home/725776/description#description

64.	Hancox JE, Quested E, Duda JL. Suitability of the Perceived Motivational Climate in Sport Questionnaire-2 for Dance Research: A Think Aloud Approach. J Dance Med Sci [Internet]. 2015;19(4):149–62. Available from: https://ovidsp.ovid.com/ovidweb.cgi?T=JS&CSC=Y&NEWS=N&PAGE=fulltext&D=emed16&DO=10.12678%2f1089-313X.19.4.149

65.	Hanif F, Khan RA, Yasmeen R. Validation and Reliability of Hidden Informal Curriculum Assessment Tool In Pakistani Preceptive. Pakistan Armed Forces Medical Journal [Internet]. 2025;75(1):127–32. Available from: https://www.scopus.com/inward/record.uri?eid=2-s2.0-86000122631&doi=10.51253%2fpafmj.v75i1.11568&partnerID=40&md5=afbd15cd0705d72b3c19b77e65830c69

66.	Hanning RM, Royall D, Toews JE, Blashill L, Wegener J, Driezen P. Web-based food behaviour questionnaire: Validation with grades six to eight students. Canadian Journal of Dietetic Practice and Research [Internet]. 2009;70(4):172–8. Available from: http://www.metapress.com/content/5ru5474037707672/fulltext.pdf

67.	Hartmann-Boyce J, Aveyard P, Koshiaris C, Jebb SA. Development of tools to study personal weight control strategies: OxFAB taxonomy. Obesity [Internet]. 2016;24(2):314–20. Available from: http://onlinelibrary.wiley.com/journal/10.1002/(ISSN)1930-739X

68.	Hartmann-Boyce J, Harmer G, Hobson A, Bateman PA, Tudor K, Aveyard P, et al. Oxford Food and Activity Behaviors 20-item questionnaire to assess personal weight management strategies: Development and testing. Obesity [Internet]. 2022;30(9):1752–9. Available from: http://onlinelibrary.wiley.com/journal/10.1002/(ISSN)1930-739X

69.	Hauge CH, Jacobs-Knight J, Jensen JL, Burgess KM, Puumala SE, Wilton G, et al. Establishing survey validity and reliability for American Indians through “think aloud” and test-retest methods. Qual Health Res [Internet]. 2015;25(6):820–30. Available from: https://ovidsp.ovid.com/ovidweb.cgi?T=JS&CSC=Y&NEWS=N&PAGE=fulltext&D=emed16&DO=10.1177%2f1049732315582010

70.	Hébert R, Raîche M, Gueye NR. Survey disability questionnaire does not generate valid accurate data compared to clinical assessment on an older population. Arch Gerontol Geriatr [Internet]. 2012;54(2):e57–62. Available from: https://www.scopus.com/inward/record.uri?eid=2-s2.0-84857059896&doi=10.1016%2fj.archger.2011.06.021&partnerID=40&md5=c4b2868292f3101aacde53cc92605903

71.	Hennel EK, Subotic U, Berendonk C, Stricker D, Harendza S, Huwendiek S. A german-language competency-based multisource feedback instrument for residents: development and validity evidence. BMC Med Educ [Internet]. 2020;20(1):357. Available from: https://ovidsp.ovid.com/ovidweb.cgi?T=JS&CSC=Y&NEWS=N&PAGE=fulltext&D=emed21&DO=10.1186%2fs12909-020-02259-2

72.	Hennrich P, Arnold C, Koetsenruijter J, Wensing M. Measuring continuity of ambulatory cardiovascular care: a cross-sectional study on the applicability of the Nijmegen Continuity Questionnaire in Germany. BMC Health Serv Res [Internet]. 2022;22(1):1258. Available from: https://ovidsp.ovid.com/ovidweb.cgi?T=JS&CSC=Y&NEWS=N&PAGE=fulltext&D=emed24&DO=10.1186%2fs12913-022-08612-z

73.	Henry G, Butow P, Juraskova I, Laidsaar-Powell R, Shaw J. Measuring female breast cancer survivors’ concerns about late effects of treatment: initial development of the Concerns about Late Effects in Oncology Questionnaire (CLEO). Supportive Care in Cancer [Internet]. 2022;30(4):3563–72. Available from: https://link.springer.de/link/service/journals/00520/index.htm

74.	Hernan AL, Giles SJ, O’Hara JK, Fuller J, Johnson JK, Dunbar JA. Developing a primary care patient measure of safety (PC PMOS): A modified Delphi process and face validity testing. BMJ Qual Saf [Internet]. 2016;25(4):273–80. Available from: http://qualitysafety.bmj.com/content/25/4/273.full.pdf

75.	Hildingsson I, Rubertsson C, Karlström A, Haines H. Exploring the Fear of Birth Scale in a mixed population of women of childbearing age—A Swedish pilot study. Women and Birth. 2018 Oct 1;31(5):407–13.

76.	Hoffmann MD, Loughead TM. Preliminary development of a questionnaire to assess peer athlete mentoring functions: the Athlete Mentoring Questionnaire (AMQ). Meas Phys Educ Exerc Sci [Internet]. 2019;23(1):10–25. Available from: http://www.tandfonline.com/toc/hmpe20/current

77.	Hornsey S, Stuart B, Muller I, Layton AM, Morrison L, King J, et al. Patient-reported outcome measures for acne: A mixed-methods validation study (acne PROMs). BMJ Open [Internet]. 2021;11(3). Available from: https://www.scopus.com/inward/record.uri?eid=2-s2.0-85103030738&doi=10.1136%2fbmjopen-2019-034047&partnerID=40&md5=7c531d5388b01b2077f1f8f8be824162

78.	Horwood J, Sutton E, Coast J. Evaluating the Face Validity of the ICECAP-O Capabilities Measure: A “Think Aloud” Study with Hip and Knee Arthroplasty Patients. Appl Res Qual Life [Internet]. 2014;9(3):667–82. Available from: https://www.scopus.com/inward/record.uri?eid=2-s2.0-84905197915&doi=10.1007%2fs11482-013-9264-4&partnerID=40&md5=70feda57352a6c31ddb819a7ca58104a

79.	Horwood J, Pollard B, Ayis S, McIlvenna T, Johnston M. Listening to patients: Using verbal data in the validation of the Aberdeen Measures of Impairment, Activity Limitation and Participation Restriction (Ab-IAP). BMC Musculoskelet Disord [Internet]. 2010;11. Available from: https://www.scopus.com/inward/record.uri?eid=2-s2.0-77955382376&doi=10.1186%2f1471-2474-11-182&partnerID=40&md5=cb36fdbbde2df09594ae0ac3bf4d55e5

80.	Hudon E, M.-C. C, Krieg C, Lambert M, Joober H, Lawn S, et al. The French adaptation and validation of the Partners in Health (PIH) scale among patients with chronic conditions seen in primary care. PLoS One [Internet]. 2019;14(10):e0224191. Available from: https://journals.plos.org/plosone/article/file?id=10.1371/journal.pone.0224191&type=printable

81.	Humphrey L, Kulich K, Deschaseaux C, Blackburn S, Maguire L, Strömberg A. The Caregiver Burden Questionnaire for Heart Failure (CBQ-HF): Face and content validity. Health Qual Life Outcomes. 2013 May 25;11(1).

82.	Huusko L, Sjöberg S, Ekström A, Hertfelt Wahn E, Thorstensson S. First-Time Fathers’ Experience of Support from Midwives in Maternity Clinics: An Interview Study. Nurs Res Pract. 2018 Nov 8;2018:1–7.

83.	Igwesi-Chidobe CN, Sorinola IO, Ozumba BC, Godfrey EL. Challenges of Measuring Self-Reported Exposure to Occupational Biomechanical Risk Factors Amongst People with Low Literacy Engaged in Manual Labour: Findings from a Cross-Cultural Adaptation and Psychometric Investigation in an African Population with Chronic Low Back Pain. J Occup Rehabil [Internet]. 2024;34(4):847–62. Available from: https://ovidsp.ovid.com/ovidweb.cgi?T=JS&CSC=Y&NEWS=N&PAGE=fulltext&D=emed27&DO=10.1007%2fs10926-024-10171-5

84.	Igwesi-Chidobe CN, Sorinola IO, Godfrey EL. Only two subscales of the Coping Strategies Questionnaire are culturally relevant for people with chronic low back pain in Nigerian Igbo populations: a cross-cultural adaptation and validation study. J Patient Rep Outcomes [Internet]. 2021;5(1):85. Available from: https://ovidsp.ovid.com/ovidweb.cgi?T=JS&CSC=Y&NEWS=N&PAGE=fulltext&D=emed27&DO=10.1186%2fs41687-021-00367-1

85.	Iqbal MZ, Ivan R, Robb C, Derby J. Evaluating factors that impact scoring an open response situational judgment test: a mixed methods approach. Front Med (Lausanne) [Internet]. 2024;11:1525156. Available from: https://journal.frontiersin.org/journal/medicine

86.	Jeannis H, Goldberg M, Seelman K, Schmeler M, Cooper RA. Participation in science and engineering laboratories for students with physical disabilities: survey development and psychometrics. Disabil Rehabil Assist Technol [Internet]. 2019;14(7):692–709. Available from: https://ovidsp.ovid.com/ovidweb.cgi?T=JS&CSC=Y&NEWS=N&PAGE=fulltext&D=emed20&DO=10.1080%2f17483107.2018.1499049

87.	Jefferson L, Brealey S, Handoll H, Keding A, Kottam L, Sbizzera I, et al. Impact of the PROFHER trial findings on surgeons’ clinical practice an online questionnaire survey. Bone Joint Res [Internet]. 2017;6(10):590–9. Available from: http://bjr.boneandjoint.org.uk/content/6/10/590.full-text.pdf

88.	Jeffrey NK, Senn CY. Gender Differences in Sexual Violence Victimization Experiences and Validity of Victimization Reports: A Think-Aloud Study. J Sex Res [Internet]. 2024;1–17. Available from: https://ovidsp.ovid.com/ovidweb.cgi?T=JS&CSC=Y&NEWS=N&PAGE=fulltext&D=emed27&DO=10.1080%2f00224499.2024.2397496

89.	Kabboord AD, M van E, L van D, Wouters M, Koet M, R van B, et al. Reliability and usability of a weighted version of the functional comorbidity index. Clin Interv Aging [Internet]. 2019;14:289–99. Available from: https://www.dovepress.com/getfile.php?fileID=47967

90.	Kaklamanou D, Armitage CJ, Jones CR. A further look into compensatory health beliefs: a think aloud study. Br J Health Psychol [Internet]. 2013;18(1):139–54. Available from: https://ovidsp.ovid.com/ovidweb.cgi?T=JS&CSC=Y&NEWS=N&PAGE=fulltext&D=emed14&AN=369254847

91.	Kaltenbrunner M, Bengtsson L, Mathiassen SE, Engstrom M. A questionnaire measuring staff perceptions of Lean adoption in healthcare: development and psychometric testing. BMC Health Serv Res [Internet]. 2017;17(1):235. Available from: https://ovidsp.ovid.com/ovidweb.cgi?T=JS&CSC=Y&NEWS=N&PAGE=fulltext&D=emed18&DO=10.1186%2fs12913-017-2163-x

92.	Kaltenbrunner M, Hagerman H, Fagerstrom C, Hartveit M, Nordheim E, Ekstedt M. The Implementation Process Assessment Tool: translation, contextualization, and psychometric evaluation of a Swedish version in a municipal elderly care context. BMC Health Serv Res [Internet]. 2024;24(1):1391. Available from: https://ovidsp.ovid.com/ovidweb.cgi?T=JS&CSC=Y&NEWS=N&PAGE=fulltext&D=emed27&DO=10.1186%2fs12913-024-11889-x

93.	Karimijashni M, Westby M, Ramsay T, Beaule PE, Poitras S. Development and content validation of a questionnaire identifying patients’ functional priorities and abilities after hip or knee arthroplasty. Disabil Rehabil [Internet]. 2024;1–15. Available from: https://ovidsp.ovid.com/ovidweb.cgi?T=JS&CSC=Y&NEWS=N&PAGE=fulltext&D=emed27&DO=10.1080%2f09638288.2024.2390648

94.	Kassam A, Douglas ML, Simon J, Cunningham S, Fassbender K, Shaw M, et al. Behaviors in Advance Care Planning and ACtions Survey (BACPACS): Development and validation part 1. BMC Palliat Care [Internet]. 2017;16(1):56. Available from: http://www.biomedcentral.com/bmcpalliatcare/

95.	Khanna D, Lay K, Khadka J, Mpundu-Kaambwa C, Ratcliffe J. How do children understand and respond to the EQ-5D-Y-3L? A mixed methods study in a community-based sample of 6-12-year-olds. Health Qual Life Outcomes [Internet]. 2024;22(1):105. Available from: https://hqlo.biomedcentral.com

96.	Kiejna A, Jakubczyk MH, Gondek TM, Rajba B. The Staff Attitude to Coercion Scale (SACS) - Polish adaptation. Psychiatr Pol [Internet]. 2020;54(1):113–24. Available from: http://psychiatriapolska.pl/uploads/images/PP_1_2020/ENGver113Kiejna_PsychiatrPol2020v54i1.pdf

97.	Kovacevic Lepojevic M, Radanovic A, Trajkovic M, Mijatovic L, Micic I. Short Form of Mental Health Literacy Questionnaire for Adults: Psychometric Properties of Serbian Version. Inquiry (United States) [Internet]. 2024;61. Available from: https://www.scopus.com/inward/record.uri?eid=2-s2.0-85205152164&doi=10.1177%2f00469580241274031&partnerID=40&md5=dceda0ee178059a1f8239d5642afb797

98.	Krieger B, Schulze C, Boyd J, Amann R, Piskur B, Beurskens A, et al. Cross-cultural adaptation of the Participation and Environment Measure for Children and Youth (PEM-CY) into German: a qualitative study in three countries. BMC Pediatr [Internet]. 2020;20(1):492. Available from: http://www.biomedcentral.com/bmcpediatr/

99.	Krieger B, Ederer F, Amann R, Morgenthaler T, Schulze C, Dawal B. Translation and cross-cultural adaptation of the young children participation and environment measure for its use in Austria, Germany, and Switzerland. Front Pediatr [Internet]. 2023;11. Available from: https://www.scopus.com/inward/record.uri?eid=2-s2.0-85182423423&doi=10.3389%2ffped.2023.1258377&partnerID=40&md5=ea5544d0d4cbf4cac836f26a2d0d0c78

100.	Krishna-Naik V, Palmer A, Hodson NA, Tugnait A, O’Connor DB. Utilisation of a think-aloud protocol to validate a self-reported periodontitis questionnaire. J Dent [Internet]. 2024;150:105381. Available from: https://ovidsp.ovid.com/ovidweb.cgi?T=JS&CSC=Y&NEWS=N&PAGE=fulltext&D=emed27&DO=10.1016%2fj.jdent.2024.105381

101.	Laberge M, Heykoop C, Camp PG. Personal disaster preparedness of people with chronic obstructive pulmonary disease: Development and validation of a self-report questionnaire. Canadian Journal of Respiratory, Critical Care, and Sleep Medicine [Internet]. 2021;5(5):316–22. Available from: https://www.tandfonline.com/toc/ucts20/current

102.	Lay K, Crocker M, Engel L, Ratcliffe J, Milte R, Hutchinson C. Exploring older people’s understanding of the QOL-ACC, a new preference-based quality-of-life measure, for quality assessment and economic evaluation in aged care: the impact of cognitive impairment and dementia. Health Qual Life Outcomes [Internet]. 2024;22(1):4. Available from: https://hqlo.biomedcentral.com

103.	Loof H, U.-B. J, Henriksson EW, Lindblad S, Saboonchi F. Development and psychometric testing of the Swedish version of the Body Awareness Questionnaire. J Adv Nurs [Internet]. 2013;69(7):1643–51. Available from: https://ovidsp.ovid.com/ovidweb.cgi?T=JS&CSC=Y&NEWS=N&PAGE=fulltext&D=emed14&DO=10.1111%2fjan.12020

104.	Manns S, Cramp F, Lewis R, Clark EM, Palmer S. A qualitative evaluation of the appropriateness, validity, acceptability, feasibility and interpretability of the Bristol Impact of Hypermobility (BIoH) questionnaire. Musculoskelet Sci Pract [Internet]. 2018;38:69–76. Available from: https://www.journals.elsevier.com/musculoskeletal-science-and-practice

105.	Marconi E, F BB, Meldolesi E, Mariani S, Panza G, Nardangeli A, et al. The Italian version of the Pediatric Quality of Life InventoryTM (PEDSQLTM) 3.0 healthcare satisfaction hematology/oncology module: reliability and validity in radiation oncology. Health Qual Life Outcomes [Internet]. 2023;21(1):72. Available from: https://hqlo.biomedcentral.com

106.	Marmura H, Bryant DM, Hansen CF, Brodersen JB, Krogsgaard M, Dhanoya S, et al. English Translation and Cultural Adaptation of the Knee Numeric-Entity Evaluation Score (KNEES-ACL): A Condition-Specific Patient-Reported Outcome Measure for Anterior Cruciate Ligament Injuries. Am J Sports Med [Internet]. 2024;52(12):2980–6. Available from: https://ovidsp.ovid.com/ovidweb.cgi?T=JS&CSC=Y&NEWS=N&PAGE=fulltext&D=emed27&DO=10.1177%2f03635465241274151

107.	Martin S, Shah SN, Hepp Z, Harris N, Morgans AK. Qualitative Analysis of Pain in Patients With Locally Advanced or Metastatic Urothelial Carcinoma. Bladder Cancer [Internet]. 2022;8(1):45–53. Available from: http://www.iospress.nl/journal/bladder-cancer/

108.	Mayland CR, Lees C, Germain A, Jack BA, Cox TF, Mason SR, et al. Caring for those who die at home: The use and validation of “Care Of the Dying Evaluation” (CODE) with bereaved relatives. BMJ Support Palliat Care [Internet]. 2014;4(2):167–74. Available from: https://www.scopus.com/inward/record.uri?eid=2-s2.0-84936989570&doi=10.1136%2fbmjspcare-2013-000596&partnerID=40&md5=2572496df549da37b88f1d65c3d9cf64

109.	McBurnie J, Bell C, Hurst N, Chambers S, Graham-Wisener L, Toner P. Content validity of the post-traumatic growth inventory: a think-aloud study on capturing recovery from addiction. Addiction Research and Theory [Internet]. 2024;32(4):255–63. Available from: https://www.scopus.com/inward/record.uri?eid=2-s2.0-85171283022&doi=10.1080%2f16066359.2023.2256217&partnerID=40&md5=8c6221980576480c17e6da04e60c90e2

110.	McClam M, Workman L, Dias EM, Walker TJ, Brandt HM, Craig DW, et al. Using cognitive interviews to improve a measure of organizational readiness for implementation. BMC Health Serv Res [Internet]. 2023;23(1):93. Available from: https://ovidsp.ovid.com/ovidweb.cgi?T=JS&CSC=Y&NEWS=N&PAGE=fulltext&D=emed26&DO=10.1186%2fs12913-022-09005-y

111.	McCorry NK, Scullion L, McMurray CM, Houghton R, Dempster M. Content validity of the illness perceptions questionnaire - revised among people with type 2 diabetes: A think-aloud study. Psychol Health [Internet]. 2013;28(6):675–85. Available from: https://ovidsp.ovid.com/ovidweb.cgi?T=JS&CSC=Y&NEWS=N&PAGE=fulltext&D=emed14&DO=10.1080%2f08870446.2012.746690

112.	McLoughlin C, Goranitis I, Al-Janabi H. The Feasibility and Validity of Preference-Based Quality of Life Measures With Informal Carers: A Think-Aloud Study. Value in Health [Internet]. 2023;26(11):1655–64. Available from: http://www.elsevier.com/wps/find/journaldescription.cws_home/724501/description#description

113.	Medina-Porqueres I, Velazquez DR, Torrecilla FM, Orava S, Cacchio A. Translation, Linguistic Validation, and Readability of the Spanish Version of the VISA-H Scale in Elite Athletes. Archives of Bone and Joint Surgery [Internet]. 2022;10(8):683–94. Available from: https://abjs.mums.ac.ir/article_19361.html

114.	Mertens VC, Moser A, Verbunt J, Smeets R, Goossens M. Content Validity of the Credibility and Expectancy Questionnaire in a Pain Rehabilitation Setting. Pain Practice. 2017 Sep 1;17(7):902–13.

115.	Mills S, Tuffrey C, Tbaily L, Tighe M. Modification of the Paediatric Gastro-oesophageal Reflux Disease Symptom and Quality of Life Questionnaire (PGSQ) for children with cerebral palsy: a preliminary study. BMJ Paediatr Open [Internet]. 2024;8(1):e002256. Available from: http://bmjpaedsopen.bmj.com/

116.	Mitchell PM, Caskey FJ, Scott J, Sanghera S, Coast J. Response process validity of three patient reported outcome measures for people requiring kidney care: A think-aloud study using the EQ-5D-5L, ICECAP-A and ICECAP-O. BMJ Open [Internet]. 2020;10(5):e034569. Available from: http://bmjopen.bmj.com/content/early/by/section

117.	Mitrofanova E, Pummell E, Martinelli L, Petroczi A. Does ORTO-15 produce valid data for “Orthorexia Nervosa”? A mixed-method examination of participants’ interpretations of the fifteen test items. Eating and Weight Disorders [Internet]. 2021;26(3):897–909. Available from: http://link.springer.com/journal/40519

118.	Mohr W, Radke A, Michalowsky B, Hoffmann W. Elicitation of quantitative, choice-based preferences for Person-Centered Care among People living with Dementia in comparison to physicians’ judgements in Germany: study protocol for the mixed-methods PreDemCare-study. BMC Geriatr [Internet]. 2022;22(1):567. Available from: https://ovidsp.ovid.com/ovidweb.cgi?T=JS&CSC=Y&NEWS=N&PAGE=fulltext&D=emed24&DO=10.1186%2fs12877-022-03238-6

119.	Monaca C, Weigl M, Pfaff H, Hammer A. Safety Culture Through Patient Voice: Qualitative Validation of the Patients’ Perceptions of Safety Culture Scale (PaPSC) in Cardiology and Cardiothoracic Surgery. Health Expectations [Internet]. 2025;28(2). Available from: https://www.scopus.com/inward/record.uri?eid=2-s2.0-86000652537&doi=10.1111%2fhex.70213&partnerID=40&md5=02bdf627ee8efb390851beea9103dcaf

120.	Monahan PO, Kroenke K, Callahan CM, Bakas T, Harrawood A, Lofton P, et al. Development and Feasibility of SymTrak, aMulti-domain Tool for Monitoring Symptoms of Older Adults in Primary Care. J Gen Intern Med [Internet]. 2019;34(6):915–22. Available from: http://www.springerlink.com/content/120414/

121.	Moodley J, Scott SE, Mwaka AD, Constant D, Githaiga JN, Stewart TS, et al. Development and validation of the african women awareness of CANcer (AWACAN) tool for breast and cervical cancer. PLoS One [Internet]. 2019;14(8):e0220545. Available from: https://journals.plos.org/plosone/article/file?id=10.1371/journal.pone.0220545&type=printable

122.	Murphy M, Hollinghurst S, Salisbury C. Qualitative assessment of the primary care outcomes questionnaire: A cognitive interview study. BMC Health Serv Res. 2018 Feb 1;18(1).

123.	Murtagh FEM, Addington-Hall JM, Higginson IJ. The value of cognitive interviewing techniques in palliative care research. Palliat Med [Internet]. 2007;21(2):87–93. Available from: https://ovidsp.ovid.com/ovidweb.cgi?T=JS&CSC=Y&NEWS=N&PAGE=fulltext&D=emed10&DO=10.1177%2f0269216306075367

124.	Muthu MS, Ganesh A, Padmanabhan R, Nuvvula S, Mohanraj R. Development and validation of a scale to assess Feeding at Sleep Time (FeAST) among infants and toddlers. Community Dent Oral Epidemiol [Internet]. 2020;48(6):471–9. Available from: https://www.scopus.com/inward/record.uri?eid=2-s2.0-85088105423&doi=10.1111%2fcdoe.12558&partnerID=40&md5=17277c218e118b3b221626c6659c16de

125.	Nafees Z, Ferreira J, Guadagno E, Wray J, Anderzen-Carlsson A, Poenaru D. Adaptation, Translation, and Validation of a Patient-Reported Experience Measure for Children and Young People for the Canadian Context. J Pediatr Surg [Internet]. 2024;59(5):810–7. Available from: https://www.sciencedirect.com/science/journal/00223468

126.	Namisango E, Luyirika EBK, Berger A. Cross Cultural Adaptation and Cognitive Testing of a Psycho-Social-Spiritual Healing Measure, the NIH Healing Experiences in All Life Stressors-NIH-HEALS. Glob Adv Health Med. 2022 Jan 19;11.

127.	Ngorsuraches S, Lerkiatbundit S, Li SC, Treesak C, Sirithorn R, Korwiwattanakarn M. Development and validation of the patient trust in community pharmacists (TRUST-Ph) scale: Results from a study conducted in Thailand. Research in Social and Administrative Pharmacy [Internet]. 2008;4(3):272–83. Available from: https://ovidsp.ovid.com/ovidweb.cgi?T=JS&CSC=Y&NEWS=N&PAGE=fulltext&D=emed10&DO=10.1016%2fj.sapharm.2007.10.002

128.	Nielsen MG, Lyng KD, Holden S, Johansen SK, Winters M, Rathleff MS. Item generation for a new patient-reported outcome measure: The non-traumatic anterior knee pain (AKP)-YOUTH scale. Musculoskelet Sci Pract [Internet]. 2024;73:103151. Available from: https://www.sciencedirect.com/science/journal/24687812

129.	Nikolaus CJ, Ellison B, Nickols-Richardson SM. College students’ interpretations of food security questions: results from cognitive interviews. BMC Public Health [Internet]. 2019;19(1):1282. Available from: https://ovidsp.ovid.com/ovidweb.cgi?T=JS&CSC=Y&NEWS=N&PAGE=fulltext&D=emed20&DO=10.1186%2fs12889-019-7629-9

130.	K NK, Boe H, Johansson E, Henriksen E, McCormack B, Gustavsson JP, et al. Swedish translation, adaptation and psychometric evaluation of the Context Assessment Index (CAI). Worldviews on evidence-based nursing / Sigma Theta Tau International, Honor Society of Nursing [Internet]. 2013;10(1):41–50. Available from: https://ovidsp.ovid.com/ovidweb.cgi?T=JS&CSC=Y&NEWS=N&PAGE=fulltext&D=emed14&DO=10.1111%2fj.1741-6787.2012.00252.x

131.	Nymark C, Saboonchi F, Mattiasson AC, Henriksson P, Kiessling A. Development and validation of an instrument to assess patients’ appraisal, emotions and action tendencies preceding care-seeking in acute myocardial infarction: The PA-AMI questionnaire. European Journal of Cardiovascular Nursing. 2017 Mar 1;16(3):240–8.

132.	Olsson E, Anderzen-Carlsson A, Atladottir SM, Axelin A, Campbell-Yeo M, Eriksson M, et al. Cultural adaptation and harmonization of four Nordic translations of the revised Premature Infant Pain Profile (PIPP-R). BMC Pediatr [Internet]. 2018;18(1):349. Available from: http://www.biomedcentral.com/bmcpediatr/

133.	Oosterveld-Vlug MG, Pasman HRW, E van GI, W de VHC, Onwuteaka-Philipsen BD. Assessing the validity and intra-observer agreement of the MIDAM-LTC; an instrument measuring factors that influence personal dignity in long-term care facilities. Health Qual Life Outcomes [Internet]. 2014;12(1):17. Available from: http://www.hqlo.com/content/12/1/17

134.	Ossenberg C, Dalton M, Henderson A. Validation of the Australian Nursing Standards Assessment Tool (ANSAT): A pilot study. Nurse Educ Today [Internet]. 2016;36:23–30. Available from: https://ovidsp.ovid.com/ovidweb.cgi?T=JS&CSC=Y&NEWS=N&PAGE=fulltext&D=emed17&DO=10.1016%2fj.nedt.2015.07.012

135.	Paige SR, Stellefson M, Krieger JL, Miller MD, Cheong J, Anderson-Lewis C. Transactional eHealth Literacy: Developing and Testing a Multi-Dimensional Instrument. J Health Commun [Internet]. 2019;24(10):737–48. Available from: https://ovidsp.ovid.com/ovidweb.cgi?T=JS&CSC=Y&NEWS=N&PAGE=fulltext&D=emed20&DO=10.1080%2f10810730.2019.1666940

136.	Palmer S, Cramp F, Lewis R, Gould G, Clark EM. Development and initial validation of the Bristol Impact of Hypermobility questionnaire. Physiotherapy (United Kingdom) [Internet]. 2017;103(2):186–92. Available from: https://ovidsp.ovid.com/ovidweb.cgi?T=JS&CSC=Y&NEWS=N&PAGE=fulltext&D=emed18&DO=10.1016%2fj.physio.2016.04.002

137.	Pandina G, Ness S, Trudeau J, Stringer S, Knoble N, Lenderking WR, et al. Qualitative evaluation of the Autism Behavior Inventory: use of cognitive interviewing to establish validity of a caregiver report scale for autism spectrum disorder. Health Qual Life Outcomes [Internet]. 2021;19(1):26. Available from: http://www.hqlo.com/home/

138.	Panepinto JA, Torres S, Varni JW. Development of the PedsQLTM Sickle Cell Disease Module items: qualitative methods. Qual Life Res [Internet]. 2012;21(2):341–57. Available from: https://ovidsp.ovid.com/ovidweb.cgi?T=JS&CSC=Y&NEWS=N&PAGE=fulltext&D=emed13&AN=366392982

139.	Park E, Kwon M. Testing the digital health literacy instrument for adolescents: Cognitive interviews. J Med Internet Res [Internet]. 2021;23(3):e17856. Available from: https://www.jmir.org/2021/3/e17856/PDF

140.	Paterson H, Hay-Smith J, Treharne G, Herbison P, Howarth C. Validation of the Intuitive Eating Scale in pregnancy. J Health Psychol [Internet]. 2018;23(5):701–9. Available from: https://ovidsp.ovid.com/ovidweb.cgi?T=JS&CSC=Y&NEWS=N&PAGE=fulltext&D=emed19&DO=10.1177%2f1359105316671186

141.	Peitz D, Schulze J, Warschburger P. Getting a deeper understanding of mindfulness in the context of eating behavior: Development and validation of the Mindful Eating Inventory. Appetite [Internet]. 2021;159:105039. Available from: http://www.elsevier.com/inca/publications/store/6/2/2/7/8/5/index.htt

142.	Peled AW, Amara D, Piper ML, Klassen AF, Tsangaris E, Pusic AL. Development and Validation of a Nipple-Specific Scale for the BREAST-Q to Assess Patient-Reported Outcomes following Nipple-Sparing Mastectomy. Plast Reconstr Surg [Internet]. 2019;143(4):1010–7. Available from: https://ovidsp.ovid.com/ovidweb.cgi?T=JS&CSC=Y&NEWS=N&PAGE=fulltext&D=emed20&DO=10.1097%2fPRS.0000000000005426

143.	Penton H, Dayson C, Hulme C, Young T. A Qualitative Investigation of Older Adults’ Conceptualization of Quality of Life and a Think-Aloud Content Validation of the EQ-5D-5L, SF-12v2, Warwick Edinburgh Mental Wellbeing Scale, and Office of National Statistics-4. Value in Health. 2022 Dec 1;25(12):2017–27.

144.	Järemo P, Arman M. Translation and cultural adaptation of the Illness Invalidation Inventory for use in Sweden. BMC Psychol. 2014 Dec;2(1).

145.	Pitulaj A, Rajba B, Andrzejewska B, Kiejna A, Dominiak M. Psychometric validation of Corah’s Dental Anxiety Scale in the Polish population. Advances in Clinical and Experimental Medicine [Internet]. 2020;29(1):45–9. Available from: http://www.advances.umed.wroc.pl/pdf/2020/29/1/45.pdf

146.	Plazar D, Metyovinyi Z, Medvecz M, Rencz F. Qualitative evidence on EQ-5D-5L skin irritation and self-confidence bolt-ons in Darier’s disease and Hailey-Hailey disease. Quality of Life Research [Internet]. 2024; Available from: https://www.springer.com/journal/11136

147.	Pool JJM, Hiralal SR, Ostelo RWJG, van der Veer K, de Vet HCW. Added value of qualitative studies in the development of health related patient reported outcomes such as the pain coping and cognition list in patients with sub-acute neck pain. Man Ther. 2010 Feb;15(1):43–7.

148.	Prebble KS, Gerbild H, Abrahamsen C. Content validity and reliability of the danish version of health care students’ attitudes towards addressing sexual health: a psychometric study. Scand J Caring Sci [Internet]. 2022;36(2):515–23. Available from: https://ovidsp.ovid.com/ovidweb.cgi?T=JS&CSC=Y&NEWS=N&PAGE=fulltext&D=emed23&DO=10.1111%2fscs.13048

149.	Prior ME, Hamzah JC, Francis JJ, Ramsay CR, Castillo MM, Campbell SE, et al. Pre-validation methods for developing a patient reported outcome instrument. BMC Med Res Methodol [Internet]. 2011;11:112. Available from: https://ovidsp.ovid.com/ovidweb.cgi?T=JS&CSC=Y&NEWS=N&PAGE=fulltext&D=emed12&AN=560021371

150.	Quandt SA, Verhoef MJ, Arcury TA, Lewith GT, Steinsbekk A, Kristoffersen AE, et al. Development of an International Questionnaire to Measure Use of Complementary and Alternative Medicine (I-CAM-Q).

151.	Rand S, Caiels J, Collins G, Forder J. Developing a proxy version of the Adult social care outcome toolkit (ASCOT). Health Qual Life Outcomes. 2017 May 19;15(1).

152.	Renberg T, A KL, Tully MP. Testing the validity of a translated pharmaceutical therapy-related quality of life instrument, using qualitative “think aloud” methodology. J Clin Pharm Ther [Internet]. 2008;33(3):279–87. Available from: https://ovidsp.ovid.com/ovidweb.cgi?T=JS&CSC=Y&NEWS=N&PAGE=fulltext&D=emed10&DO=10.1111%2fj.1365-2710.2008.00921.x

153.	Rencz F, Mukuria C, Bato A, Poor AK, Finch AP. A qualitative investigation of the relevance of skin irritation and self-confidence bolt-ons and their conceptual overlap with the EQ-5D in patients with psoriasis. Quality of Life Research [Internet]. 2022;31(10):3049–60. Available from: https://www.wkap.nl/journalhome.htm/0962-9343

154.	Riekert KA, Ford C, Goodman A, Eckmann T, Green A, Quittner AL. Supporting adherence to the cystic fibrosis regimen: Development and validation of The Daily Care Check-In (DCC). Journal of Cystic Fibrosis. 2025 Mar 1;24(2):326–33.

155.	Rodrigues AM, Jacinto S, Henriques AR, Valada MP, Tinazzi I, de Sousa RD, et al. Linguistic and cultural adaptation of the EARP Questionnaire to European Portuguese. ARP Rheumatology [Internet]. 2022;1(4):271–7. Available from: https://www.scopus.com/inward/record.uri?eid=2-s2.0-85152390499&partnerID=40&md5=5c3eb86ff71d8668ebe778ac5411915a

156.	Rodrigues PFS, Pandeirada JNS, Marinho PI, Bem-Haja P, Silva CF, Ribeiro L, et al. Morningness-eveningness preferences in Portuguese adolescents: Adaptation and psychometric validity of the H&O questionnaire. Pers Individ Dif [Internet]. 2016;88:62–5. Available from: https://www.scopus.com/inward/record.uri?eid=2-s2.0-84941253983&doi=10.1016%2fj.paid.2015.08.048&partnerID=40&md5=71a72a37824e06b401224e5f67d19d78

157.	Rogers HJ, Gilchrist F, Marshman Z, Rodd HD, Rowen D. Selection and validation of a classification system for a child-centred preference-based measure of oral health-related quality of life specific to dental caries. J Patient Rep Outcomes [Internet]. 2020;4(1):105. Available from: https://ovidsp.ovid.com/ovidweb.cgi?T=JS&CSC=Y&NEWS=N&PAGE=fulltext&D=emexb&DO=10.1186%2fs41687-020-00268-9

158.	Rohr M, Brandstetter S, Plomer AS, Loss J, Kretschmer R, Apfelbacher C. A qualitative study exploring content validity and feasibility of frequently used generic health-related quality of life measures in older people with hip fracture: The patients’ perspective. Injury. 2021 Feb 1;52(2):134–41.

159.	Rotstein E, von Rosen P, Karlström S, Knutsson JE, Rose N, Forslin E, et al. Development and initial validation of a Swedish inventory to screen for symptoms of deficient perineum in women after vaginal childbirth: ‘Karolinska Symptoms After Perineal Tear Inventory.’ BMC Pregnancy Childbirth [Internet]. 2022;22(1). Available from: https://www.scopus.com/inward/record.uri?eid=2-s2.0-85135932226&doi=10.1186%2fs12884-022-04964-w&partnerID=40&md5=704b13b158bbc192da326553a8f7bf0d

160.	Sadiqi S, Lehr AM, Post MW, Dvorak MF, Kandziora F, Rajasekaran S, et al. Development of the AOSpine Patient Reported Outcome Spine Trauma (AOSpine PROST): a universal disease-specific outcome instrument for individuals with traumatic spinal column injury. European Spine Journal. 2017 May 1;26(5):1550–7.

161.	Saldaris J, Weisenberg J, Pestana-Knight E, Marsh ED, Suter B, Rajaraman R, et al. Content Validation of Clinician-Reported Items for a Severity Measure for CDKL5 Deficiency Disorder. J Child Neurol [Internet]. 2021;36(11):998–1006. Available from: http://jcn.sagepub.com/

162.	Samuelyan N, Ay P, Moon Z, Sancar M, Horne R, Okuyan B. Reliability and validity of the Turkish version of the medication practical barriers to adherence questionnaire in patients with chronic diseases. Eur J Clin Pharmacol [Internet]. 2024;80(11):1715–23. Available from: https://www.springer.com/journal/228

163.	Schildmann EK, Groeneveld EI, Denzel J, Brown A, Bernhardt F, Bailey K, et al. Discovering the hidden benefits of cognitive interviewing in two languages: The first phase of a validation study of the Integrated Palliative care Outcome Scale. Palliat Med. 2016 Jun 1;30(6):599–610.

164.	Sekhon M, Cartwright M, Francis JJ. Development of a theory-informed questionnaire to assess the acceptability of healthcare interventions. BMC Health Serv Res. 2022 Mar 1;22(1).

165.	Sheldon EM, Lillington G, Simpson K, Gibson K, Chambers L, D’Afflitto M, et al. Development of an inflammatory bowel disease (IBD) Patient-Reported Experience Measure (PREM): A patient-led consensus work and “think aloud” study for a quality improvement programme. Health Expect [Internet]. 2023;26(1):213–25. Available from: https://ovidsp.ovid.com/ovidweb.cgi?T=JS&CSC=Y&NEWS=N&PAGE=fulltext&D=emed25&DO=10.1111%2fhex.13647

166.	Sigvardsdotter E, Nilsson H, Malm A, Tinghog P, Gottvall M, Vaez M, et al. Development and preliminary validation of refugee trauma history checklist (RTHC)-a brief checklist for survey studies. Int J Environ Res Public Health [Internet]. 2017;14(10):1175. Available from: http://www.mdpi.com/1660-4601/14/10/1175/pdf

167.	Simmonds G, Tinati T, Barker M, Bishop FL. Measuring young women’s self-efficacy for healthy eating: Initial development and validation of a new questionnaire. J Health Psychol [Internet]. 2016;21(11):2503–13. Available from: https://www.scopus.com/inward/record.uri?eid=2-s2.0-84992533053&doi=10.1177%2f1359105315580464&partnerID=40&md5=94ee54780c9fbfe52a3ccae4af0df4dd

168.	Skevington SM, Dehner S, Gillison FB, McGrath EJ, Lovell CR. How appropriate is the WHOQOL-BREF for assessing the quality of life of adolescents? Psychol Health [Internet]. 2014;29(3):297–317. Available from: https://ovidsp.ovid.com/ovidweb.cgi?T=JS&CSC=Y&NEWS=N&PAGE=fulltext&D=emed15&DO=10.1080%2f08870446.2013.845668

169.	Solli R, Kvael LAH, Olsen NR, Brovold T. Evaluation of content validity and feasibility of the World Falls Guidelines’ three key questions to identify falls among older adult users of home care services in Norway. BMC Health Serv Res [Internet]. 2025;25(1):444. Available from: https://ovidsp.ovid.com/ovidweb.cgi?T=JS&CSC=Y&NEWS=N&PAGE=fulltext&D=emexb&DO=10.1186%2fs12913-025-12606-y

170.	Sopfe J, Marsh R, Ziniel SI, Klosky JL, Chow EJ, Holliman BD, et al. Evaluation of the v2.0 brief profiles for sexual function and satisfaction PROMIS in adolescent and young adult childhood cancer survivors. J Adolesc Young Adult Oncol [Internet]. 2021;10(4):418–24. Available from: http://www.liebertpub.com/products/product.aspx?pid=387

171.	Sripaew S, Fumaneeshoat O, Ingviya T. Systematic adaptation of the Thai version of the supportive and palliative care indicators tool for low-income setting (SPICT-LIS). BMC Palliat Care [Internet]. 2021;20(1):35. Available from: http://www.biomedcentral.com/bmcpalliatcare/

172.	Sussex AK, Rencz F, Gaydon M, Lloyd A, Gallop K. Exploring the content validity of the EQ-5D-5L and four bolt-ons (skin irritation, self-confidence, sleep, social relationships) in atopic dermatitis and chronic urticaria. Quality of Life Research. 2025 Apr 1;34(4):991–1002.

173.	Svantesson M, Karlsson J, Boitte P, Schildman J, Dauwerse L, Widdershoven G, et al. Outcomes of moral case deliberation--the development of an evaluation instrument for clinical ethics support (the Euro-MCD). BMC Med Ethics [Internet]. 2014;15:30. Available from: https://ovidsp.ovid.com/ovidweb.cgi?T=JS&CSC=Y&NEWS=N&PAGE=fulltext&D=emed15&DO=10.1186%2f1472-6939-15-30

174.	Sweet SN, Shi Z, Pastore O, Shaw RB, Comeau J, Gainforth HL, et al. The spinal cord injury (SCI) peer support evaluation tool: the development of a tool to assess outcomes of peer support programs within SCI community-based organizations. Spinal Cord [Internet]. 2024;62(12):690–9. Available from: https://www.nature.com/sc/

175.	Szlavicz E, Szabo A, Kinyo A, Szeiffert A, Bancsok T, Brodszky V, et al. Content validity of the EQ-5D-5L with skin irritation and self-confidence bolt-ons in patients with atopic dermatitis: a qualitative think-aloud study. Quality of Life Research [Internet]. 2024;33(1):101–11. Available from: https://www.springer.com/journal/11136

176.	Takasaki H, Handa Y. Initial development of a patient-reported outcome measure of disability due to Katakori via evaluating patient comprehensibility and comprehensiveness [Internet]. Available from: https://www.ncbi.nlm.nih.gov/pmc/journals/2193/

177.	Tatlock S, Bentley S, Arbuckle R, Abetz-Webb L, Scott J. Content validation of a caregiver diary to monitor severity and recovery of pediatric patients with respiratory syncytial virus infection. J Patient Rep Outcomes [Internet]. 2022;6(1). Available from: https://www.scopus.com/inward/record.uri?eid=2-s2.0-85132670222&doi=10.1186%2fs41687-022-00442-1&partnerID=40&md5=78cec59a2338bcca2530560ccd2a8e46

178.	Tatlock S, Grant L, Spertus JA, Khan I, Arbuckle R, Manvelian G, et al. Development and Content Validity Testing of a Patient-Reported Treatment Acceptance Measure for Use in Patients Receiving Treatment via Subcutaneous Injection. Value in Health [Internet]. 2015;18(8):1000–7. Available from: http://www.elsevier.com/wps/find/journaldescription.cws_home/724501/description#description

179.	Taylor EC, O’Neill M, Hughes LD, Moss-Morris R. An illness-specific version of the Revised Illness Perception Questionnaire in patients with atrial fibrillation (AF IPQ-R): Unpacking beliefs about treatment control, personal control and symptom triggers. Psychol Health [Internet]. 2018;33(4):499–517. Available from: https://ovidsp.ovid.com/ovidweb.cgi?T=JS&CSC=Y&NEWS=N&PAGE=fulltext&D=emed19&DO=10.1080%2f08870446.2017.1373113

180.	Ternstrom E, Hildingsson I, Haines H, Rubertsson C. Pregnant women’s thoughts when assessing fear of birth on the Fear of Birth Scale. Women and Birth [Internet]. 2016;29(3):e44–9. Available from: http://www.elsevier.com/wps/find/journaldescription.cws_home/707424/description#description

181.	Teusen C, Bühner M, Hapfelmeier A, von Schrottenberg V, Linde K, Gensichen J, et al. Development and psychometric evaluation of a questionnaire for the assessment of depression in primary care: a cross-sectional study. BMJ Open [Internet]. 2024;14(7). Available from: https://www.scopus.com/inward/record.uri?eid=2-s2.0-85199016170&doi=10.1136%2fbmjopen-2024-084102&partnerID=40&md5=34f91e760a9dab399fa61c56320f9cc9

182.	Thermaenius J, Schandl A, Sluys KP. Development and initial validation of the Swedish Family Satisfaction Intensive Care Questionnaire (SFS-ICQ). Intensive Crit Care Nurs. 2019 Feb 1;50:118–24.

183.	Thompson C, Adams J, Vidgen HA. Progressing the development of a food literacy questionnaire using cognitive interviews. Public Health Nutr [Internet]. 2022;25(7):1968–78. Available from: https://ovidsp.ovid.com/ovidweb.cgi?T=JS&CSC=Y&NEWS=N&PAGE=fulltext&D=emed27&DO=10.1017%2fS1368980021004560

184.	Thorvaldsdottir KB, Halldorsdottir S, Johnson RM, Sigurdardottir S, D SA. Adaptation of the barriers to help-seeking for trauma (BHS-TR) scale: a cross-cultural cognitive interview study with female intimate partner violence survivors in Iceland. J Patient Rep Outcomes [Internet]. 2021;5(1):22. Available from: https://ovidsp.ovid.com/ovidweb.cgi?T=JS&CSC=Y&NEWS=N&PAGE=fulltext&D=emexb&DO=10.1186%2fs41687-021-00295-0

185.	Tremblay D, Poder TG, Vasiliadis HM, Touati N, Fortin B, Lévesque L, et al. Translation and cultural adaptation of the patient self-administered financial effects (P-safe) questionnaire to assess the financial burden of cancer in french-speaking patients. Healthcare (Switzerland) [Internet]. 2020;8(4). Available from: https://www.scopus.com/inward/record.uri?eid=2-s2.0-85104336767&doi=10.3390%2fhealthcare8040366&partnerID=40&md5=1575aa71053b0aa2ae2565a6e9fd90ab

186.	Turner-Bowker DM, Saris-Baglama RN, DeRosa MA, Paulsen CA, Bransfield CP. Using qualitative research to inform the development of a comprehensive outcomes assessment for asthma. Patient [Internet]. 2009;2(4):269–82. Available from: https://ovidsp.ovid.com/ovidweb.cgi?T=JS&CSC=Y&NEWS=N&PAGE=fulltext&D=emed11&DO=10.2165%2f11313840-000000000-00000

187.	Turner-Bowker DM, Saris-Baglama RN, DeRosa MA, Paulsen CA. Cognitive testing and readability of an item bank for measuring the impact of headache on health-related quality of life. Patient [Internet]. 2012;5(2):89–99. Available from: https://ovidsp.ovid.com/ovidweb.cgi?T=JS&CSC=Y&NEWS=N&PAGE=fulltext&D=emed13&DO=10.2165%2f11592170-000000000-00000

188.	Tyler N, Planner C, Shears B, Hernan A, Panagioti M, Giles S. Developing the Resident Measure of Safety in Care Homes (RMOS): A Delphi and Think Aloud Study. Health Expect [Internet]. 2023;26(3):1149–58. Available from: https://ovidsp.ovid.com/ovidweb.cgi?T=JS&CSC=Y&NEWS=N&PAGE=fulltext&D=emed25&DO=10.1111%2fhex.13730

189.	Van Leeuwen KM, Jansen APD, Muntinga ME, Bosmans JE, Westerman MJ, Van Tulder MW, et al. Exploration of the content validity and feasibility of the EQ-5D-3L, ICECAP-O and ASCOT in older adults. BMC Health Serv Res. 2015 Dec 12;15(1).

190.	M van LK, Bosmans JE, Jansen APD, Rand SE, A.-M. T, Smith N, et al. Dutch translation and cross-cultural validation of the Adult Social Care Outcomes Toolkit (ASCOT). Health Qual Life Outcomes [Internet]. 2015;13(1):56. Available from: http://www.hqlo.com/home/

191.	M van LL, Tamminga SJ, Ravinskaya M, A de W, Hahn EA, Terwee CB, et al. Proposal to extend the PROMIS item bank v2.0 “Ability to Participate in Social Roles and Activities”: item generation and content validity. Quality of Life Research [Internet]. 2020;29(10):2851–61. Available from: https://www.wkap.nl/journalhome.htm/0962-9343

192.	van Melle MA, van Stel HF, Poldervaart JM, de Wit NJ, Zwart DLM. The transitional risk and incident questionnaire was valid and reliable for measuring transitional patient safety from the patients’ perspective. J Clin Epidemiol [Internet]. 2019;105:40–9. Available from: https://www.scopus.com/inward/record.uri?eid=2-s2.0-85054604486&doi=10.1016%2fj.jclinepi.2018.08.002&partnerID=40&md5=49deeb85ad329d22cd1c98f0d7ace8cd

193.	Van Oort L, Schröder C, French DP. What do people think about when they answer the brief illness perception questionnaire? A “think-aloud” study. Br J Health Psychol [Internet]. 2011;16(2):231–45. Available from: https://www.scopus.com/inward/record.uri?eid=2-s2.0-79954620904&doi=10.1348%2f135910710X500819&partnerID=40&md5=6af44811ac29a356088150183656bd90

194.	van Stiphout L, Hossein I, Kimman M, Whitney SL, Ayiotis A, Strupp M, et al. Development and Content Validity of the Bilateral Vestibulopathy Questionnaire. Front Neurol [Internet]. 2022;13. Available from: https://www.scopus.com/inward/record.uri?eid=2-s2.0-85127840489&doi=10.3389%2ffneur.2022.852048&partnerID=40&md5=c20c3cf0ef8c3f7d4b9c0e4bddd59b1a

195.	Varni JW, Kay MT, Limbers CA, Franciosi JP, Pohl JF. PedsQL gastrointestinal symptoms module item development: Qualitative methods. J Pediatr Gastroenterol Nutr [Internet]. 2012;54(5):664–71. Available from: https://ovidsp.ovid.com/ovidweb.cgi?T=JS&CSC=Y&NEWS=N&PAGE=fulltext&D=emed13&DO=10.1097%2fMPG.0b013e31823c9b88

196.	Vaucher P, Bischoff T, E.-A. D, Herzig L, Meystre-Agustoni G, Panese F, et al. Detecting and measuring deprivation in primary care: Development, reliability and validity of a self-reported questionnaire: The DiPCare-Q. BMJ Open [Internet]. 2012;2(1):000692. Available from: http://bmjopen.bmj.com/content/2/1/e000692.full.pdf+html

197.	Veldhuijzen G, P DJMJ, Roosen CM, Siersema PD, Drenth JPH, J VEAA. The gastrointestinal endoscopy satisfaction questionnaire captures patient satisfaction as a key quality indicator of gastrointestinal endoscopy. Eur J Gastroenterol Hepatol [Internet]. 2020;32(7):832–7. Available from: http://journals.lww.com/eurojgh/pages/default.aspx

198.	Visser M, Sealy MJ, Leistra E, Naumann E, De van der Schueren MAE, Jager-Wittenaar H. The Malnutrition Awareness Scale for community-dwelling older adults: Development and psychometric properties. Clinical Nutrition [Internet]. 2024;43(2):446–52. Available from: https://www.scopus.com/inward/record.uri?eid=2-s2.0-85181820315&doi=10.1016%2fj.clnu.2023.12.023&partnerID=40&md5=371d2437b20f2778daca0f141136386b

199.	von Scheven E, Braun M, Nahal B, Perito ER, Brakeman P, Soulsby WD, et al. Voices of Hope: Leveraging Think-Aloud Cognitive Interviews to Develop a Hope Assessment Tool for Young People Living with Chronic Health Conditions. Children [Internet]. 2024;11(11). Available from: https://www.scopus.com/inward/record.uri?eid=2-s2.0-85210366914&doi=10.3390%2fchildren11111396&partnerID=40&md5=44ea16beea48c1b7f1fe08831b96c8bc

200.	Vreeman RC, Nyandiko WM, Ayaya SO, Walumbe EG, Inui TS. Cognitive interviewing for cross-cultural adaptation of pediatric antiretroviral therapy adherence measurement items. Int J Behav Med [Internet]. 2014;21(1):186–96. Available from: https://ovidsp.ovid.com/ovidweb.cgi?T=JS&CSC=Y&NEWS=N&PAGE=fulltext&D=emed15&DO=10.1007%2fs12529-012-9283-9

201.	Wang X, Gao S, Wang T, Xue J, Yang Y, Han L, et al. Cognitive interviewing validation of the Chinese version of the neurogenic bladder symptom score. Heliyon [Internet]. 2024;10(18). Available from: https://www.scopus.com/inward/record.uri?eid=2-s2.0-85203337767&doi=10.1016%2fj.heliyon.2024.e37435&partnerID=40&md5=98c5199c9e58dfaead5b57e185c7655f

202.	Watanabe SM, Nekolaichuk CL, Beaumont C. The Edmonton Symptom Assessment System, a proposed tool for distress screening in cancer patients: Development and refinement. Psychooncology [Internet]. 2012;21(9):977–85. Available from: https://ovidsp.ovid.com/ovidweb.cgi?T=JS&CSC=Y&NEWS=N&PAGE=fulltext&D=emed13&DO=10.1002%2fpon.1996

203.	Watanabe S, Nekolaichuk C, Beaumont C, Mawani A. The Edmonton symptom assessment system-what do patients think? Supportive Care in Cancer [Internet]. 2009;17(6):675–83. Available from: https://ovidsp.ovid.com/ovidweb.cgi?T=JS&CSC=Y&NEWS=N&PAGE=fulltext&D=emed11&DO=10.1007%2fs00520-008-0522-1

204.	Wazqar DY. Arabic version of the Palliative Care Self-Efficacy Scale: Translation, adaptation, and validation. Palliat Support Care [Internet]. 2023;21(5):863–70. Available from: https://ovidsp.ovid.com/ovidweb.cgi?T=JS&CSC=Y&NEWS=N&PAGE=fulltext&D=emed25&DO=10.1017%2fS1478951522001456

205.	Wenzel L, Haker M, Heesen C, Kasper J, Kopke S, Rahn AC. Evaluating Relapse Knowledge in People with Multiple Sclerosis: A Cross-Sectional Study on the Development and Validation of the Relapse Knowledge Questionnaire. Mult Scler Relat Disord [Internet]. 2024;83:105381. Available from: https://www.sciencedirect.com/science/journal/22110348

206.	Wiklander M, Rydström LL, Ygge BM, Navér L, Wettergren L, Eriksson LE. Psychometric properties of a short version of the HIV stigma scale, adapted for children with HIV infection [Internet]. 2013. Available from: http://www.hqlo.com/content/11/1/195

207.	Wu J, Xie S, He X, Chen G, Brazier JE. The Simplified Chinese version of SF-6Dv2: translation, cross-cultural adaptation and preliminary psychometric testing. Quality of Life Research. 2020 May 1;29(5):1385–91.

208.	Zarnowiecki D, Byrne RA, Bodner GE, Bell LK, Golley RK. Improving the reporting of young children’s food intake: Insights from a cognitive interviewing study with mothers of 3-7-year old children. Nutrients [Internet]. 2020;12(6):1645. Available from: https://www.mdpi.com/2072-6643/12/6/1645/pdf

209.	Zhang J, Browne WJ. Exploring Chinese high school students’ performance and perceptions of scientific argumentation by understanding it as a three-component progression of competencies. J Res Sci Teach [Internet]. 2023;60(4):847–84. Available from: https://www.scopus.com/inward/record.uri?eid=2-s2.0-85138518847&doi=10.1002%2ftea.21819&partnerID=40&md5=8b85ca9d6140be0ec2ee11981e805538

210.	Zuger A, Fischbeck S, Weber M, Mai S. Revision of the Advanced Cancer Patients’ Distress Scale (ACPDS): A mixed-methods study among palliative patients and healthcare professionals in Germany. BMJ Open [Internet]. 2023;13(4):e066998. Available from: http://bmjopen.bmj.com/content/early/by/section
