# Supplementary material for: Thinking-aloud methodology applied to health questionnaire development and validation: a scoping review
Source: BMC Med Res Methodol. 2026 Jun 20;26:217. doi: 10.1186/s12874-026-02927-9 (PMC13640185; doi:10.1186/s12874-026-02927-9)
Supplement: Supplementary file 3 [file 12874_2026_2927_MOESM3_ESM.docx]

|  | Technique variation | **Advantages** | **Disadvantages** |
| --- | --- | --- | --- |
| Thinking- aloud methodology | Concurrent | More prone to reactivity, verbalisation focusing on the procedure. | Participants could perform worse due to the double workload. Not possible the recording of working time per task, because TA slows the process. Possible behaviour alteration. |
|  | Retrospective | Recording of working time per task | Possible decrease in reactivity. Sessions longer duration. Verbalisations focused on explanation |
| Prompting | Yes | More information retrieval. | Can introduce a form of bias on the respondent's answer. |
|  | No | Any form of bias introduced by the interviewer. | Some respondents can find thinking-aloud difficult, and they can be unable to vocalize their thoughts. |
| Probing | Yes | Add information about respondents’ thought process.  The interviewer can focus on pertinent issues. Greater control of the interview and can keep the respondent focused on the task. | Can change the thought flow and people's responses to questions. Increased need for training of the researcher.  Can lead to the creation of artificial problems. |

|  | No | Less open to the interviewer effect. Less need for interviewer training required | Less information retrieval.  TA alone can show that a problem exists but may not be able to provide any evidence to what problems are. |
| --- | --- | --- | --- |

| Sample size |  | At least seven participants (COSMIN), between 5 and 15 interviews for a testing round before testing a revision of the questionnaire in another round. Willis (2005) | | Caulton (2001) claims that a heterogeneous sample of participants affects the relationship between sample size and stability and exhaustiveness of the problems detected. |
| --- | --- | --- | --- | --- |
| Sampling | Purposive | Purposive sampling is employed to access participants who fit  inclusion criteria. | Setting rigid criteria may inadvertently result in missed perspectives. Lack of saturation may result in researchers reporting findings from scarce’ data and yield invalid findings | |
|  | Saturation | Ideally, data collection stops only when further data will not provide additional depth to the study, producing redundant information. | Time and resource consuming. The researcher’s experience can play a role in understanding when saturation is achieved. Theoretically, saturation could never be achieved, and can depends on the diversity of the sample. | |
| Analysis method | Content | Can introduce a quantitative aspect to a qualitative analysis. Content analysis may focus on latent or manifest content | | |
|  | Thematic | Incorporates latent and manifest content. Gives the possibility to a  theme to emerge not for its frequency of occurrence but on whether it represents something relevant to the research question. | | |
| Theoretical framework |  | The use of a theoretical framework can guide data collection and analysis, or to write the discussion. Theoretical frameworks may serve as maps, directing attention to a particular phenomenon and leading re searchers to paths that may otherwise be missed | Risk of overreliance on theory, which may stifle the data or lead to biased findings to make the framework fit. | |
| Coding | Yes | Good strategy to dissect the transcripts into meaningful chunks, that can be reassembled together according to patterns. | The work of segmenting and coding the transcripts can be very time consuming. Subjective process that can be very different from researcher to researcher. A great fragmentation of the transcript can cause an interpretation independent from the larger meaning. | |
|  | No | It is possible that the absence of coding could help maintain the richness, the insight and the depth that are characteristic of qualitative research. | Have to deal with a sheer volume of data that can be sometimes arduous and confusing to interpret. | |
| Coding type | Inductive | Consents the emergence and discovery of meaning from specific instances, contexts and individuals. Could be preferable when the research is purely exploratory | | |
|  | Deductive | Preferable when there is already an explanatory framework, wherein researchers expand or test the existing theory against the collected data. | | |
|  | Mixed | Can be a good approach to start an analysis from a scratch, and then add inductively more categorisations and examples. | | |
| Interviews rounds | One | Time sparing. | If the transcripts require a questionnaire revision, the new version of the instrument won’t be validated. | |
|  | More than one | Time consuming, need for a larger sample divided in two ore more groups. It can be not necessary if the questionnaire doesn't require changes. | Every new round of interview permits the content validation of the new questionnaire draft. | |
| Use of AI |  | Time saving. AI interpretations are accurate and human-like. | The structure of prompts, the specific questions made and the general model for categorizing passages significantly impacts coding fidelity. | |

**Table 3. Resume of thinking-aloud technique variations, listing advantages and disadvantages.**
